# Cross-linguistic benchmarking of NLP metrics for psychosis research

**DOI:** 10.1038/s41746-026-03053-y

**Published:** 2026-08-03

**Authors:** D. Stein, D. Bobrovskiy, M. Stede, C. Montag, S. A. Just

**Affiliations:** 1 Universität Potsdam, Potsdam, Germany; 2 JetBrains Research, Berlin, Germany; 3 Biozentrum, University of Basel, Basel, Switzerland; 4 Department of Psychiatry and Neurosciences, Campus Charité Mitte, Charité – Universitätsmedizin Berlin, Corporate Member of Freie Universität Berlin and Humboldt Universität zu Berlin, Berlin, Germany; 5 Department of Clinical Medicine, UiT The Arctic University of Norway, Tromsø, Norway

**Keywords:** Health care, Medical research, Neurology, Neuroscience

## Abstract

Language is central to psychosis, making it a key focus for both clinical evaluation and research. Many automated linguistic metrics have been developed to analyze speech in psychosis, yet few studies have compared them systematically. In this study, we benchmarked the performance and robustness of 51 natural language processing (NLP) metrics using two datasets: one with German- and one with Russian-speaking participants. Metric performance was defined as the metrics’ capacity to reflect symptom severity and distinguish individuals with psychosis from healthy controls. The best-performing metrics tended to be robust across different clinical rating scales and speech elicitation tasks. Overall, verbosity metrics (e.g., word count) outperformed most other metrics across tasks and languages. Thus, verbosity-based metrics should be used as a baseline when evaluating cross-linguistic NLP metrics that are both clinically meaningful and useful for psychosis research.

## Introduction

Mental status assessment in psychiatry has always relied on spoken or written language to infer signs of mental illness. The way a person speaks gives insight into how their mind works^1^, into underlying biological and cognitive processes, emotional states, and, in cases of significant changes in someone’s speech, can represent the onset or worsening of mental illness^2,3^. This is particularly true for psychotic disorders where, in the case of schizophrenia, disorganized speech is at the core of the illness^4–6^, and most traditional assessments of psychotic symptoms have an emphasis on linguistic symptoms^4,7^ such as impoverished or incoherent speech^8^. In recent years, Natural Language Processing (NLP) has gained traction in mental health research, and numerous studies have employed NLP techniques to model and analyze speech patterns of individuals with psychosis, trying to find linguistic markers of the illness and its course^9–11^. The potential is transformative: automated systems that integrate speech collection, automatic speech recognition, and real-time analysis of linguistic markers promise earlier and more effective intervention through automated symptom monitoring^12,13^. However, real-world implementation requires rigorous benchmarking. In this study, we therefore evaluate the performance, robustness, and generalizability of commonly used NLP markers of psychosis to accelerate their translation into clinically meaningful applications. By systematically evaluating these issues, this work provides a reference point for future NLP studies in psychosis and beyond.

Many prior NLP studies have focused on proof-of-concept demonstrations, showing that newly developed metrics can (i) differentiate individuals with psychosis from healthy controls^14–18^, (ii) correlate with clinical ratings of symptom severity^14,19^, and (iii) predict psychosis onset in high-risk individuals^20–22^. These approaches have helped establish that NLP metrics can be linked to clinically relevant phenomena.

NLP studies to date have aimed to find markers of psychosis in speech collected through various speech elicitation tasks (e.g., open questions vs. picture description tasks^18,23,24^) as well as data from electronic health records^25,26^ or social media posts^27^. Analysis of social media data specifically raises ethical concerns as users have not consented to analysis of their data for characteristics that could indicate a mental illness^28^. Therefore, we will focus on NLP analysis of speech data collected from individuals with psychosis who have provided informed consent. We also used several elicitation tasks in each dataset, as the metric performance has previously been reported to vary across different speech elicitation tasks^29^.

Numerous NLP metrics have been suggested for analysis of data from speech elicitation tasks in individuals with psychosis^11,30^. The metrics can be broadly categorized based on the speech units that they operate upon into lexical, syntactic, text-level, as well as phonetic and discursive (see Supplementary Table 1 to 10). **Lexical** metrics operate on individual words and include measures of verbosity, such as word count, and measures of lexical diversity^31,32^, as well as word-based semantic and sentiment analyses^33,34^. **Syntactic** metrics are concerned with syntactic categories and structures, including part-of-speech frequencies^20,24^, syntactic complexity^35–37^, and the use of referential devices, such as pronouns^18,38,39^. Syntactic metrics also cover such measures of verbosity as sentence length and count^38,40^. **Text-level** metrics try to work with the meaning of the entire text, capturing overall connectedness or coherence between different parts. This is most frequently achieved by using either language-model-based or graph-based representations of a text. **Language-model-based** metrics can utilize embedding vector similarity^14,19,29^, either on surprisal or perplexity scores^41,42^ or on predictive features such as next sentence probability^9,37^ or fine-tuned model predictions about group membership or symptom severity^43,44^. **Graph-based** metrics rely on either co-occurrence^45^ or semantic relations^23,39^ to construct a graph from the text and then analyze the mathematical properties of the resulting graph structure. **Phonetic** metrics focus on the spoken texts and analyze features of pronunciation and intonation^46–48^. Finally, **discursive** features include such aspects as pausation patterns, or the use of filler words^49,50^, or the change of conversation turns^51^. In our study, we focus on the lexical, syntactic, and text-level metrics.

To date, NLP research on psychosis has utilized a wide range of models, embeddings, and training data; yet approaches are rarely systematically compared against one another or examined for psychometric properties. This has prompted calls for systematic psychometric evaluation of NLP metrics – in psychosis research and beyond^3,11,19,52–54^. Such evaluation requires robustly performing, simple, and interpretable baseline metrics against which other approaches can be benchmarked. If more complex metrics cannot achieve higher performance than the selected baseline, this suggests that such metrics may need to be reconsidered as individual predictors for a specific clinical application. Markers of verbosity could play the role of such a baseline, as both reduced and increased amount of speech are prominent symptoms in psychosis^8^. In addition, the amount of speech is an important indicator of mental state, associated with the overall severity of symptoms, not merely in psychosis^18,35,37^, but across mental health conditions^55–57^, making it a potential marker of general mental and cognitive health. Systematic evaluation of NLP metrics further requires that it is conducted across different languages. A limitation of the current NLP research is that it has mostly examined speech data in English, with metrics showing a limited generalizability to other languages^10,58^. Finally, results may vary across different speech elicitation tasks^29^.

We aim to address these gaps by performing benchmarking of NLP metrics in psychosis research. Using verbosity metrics as baselines, we systematically evaluate how well other NLP metrics perform across speech elicitation tasks and two languages, namely German and Russian. We analyze the relative performance of the selected metrics in differentiating individuals with and without psychosis as well as the strength of correlations with symptom scores. We compare the performance of all metrics to those measuring verbosity, which we chose as baselines on account of their simplicity and established connection with clinical features.

The present study thus aims to answer the following questions. Firstly, how the most commonly used NLP metrics compare against each other in terms of reaching two performance indicators of NLP metrics: (i) differentiating between individuals with psychosis and healthy control subjects, and (ii) correlation with clinical ratings of symptoms. Secondly, which of these metrics outperform simple verbosity baselines, and, finally, which metrics show reliable performance between tasks and languages.

## Results

**Fig. 1:**
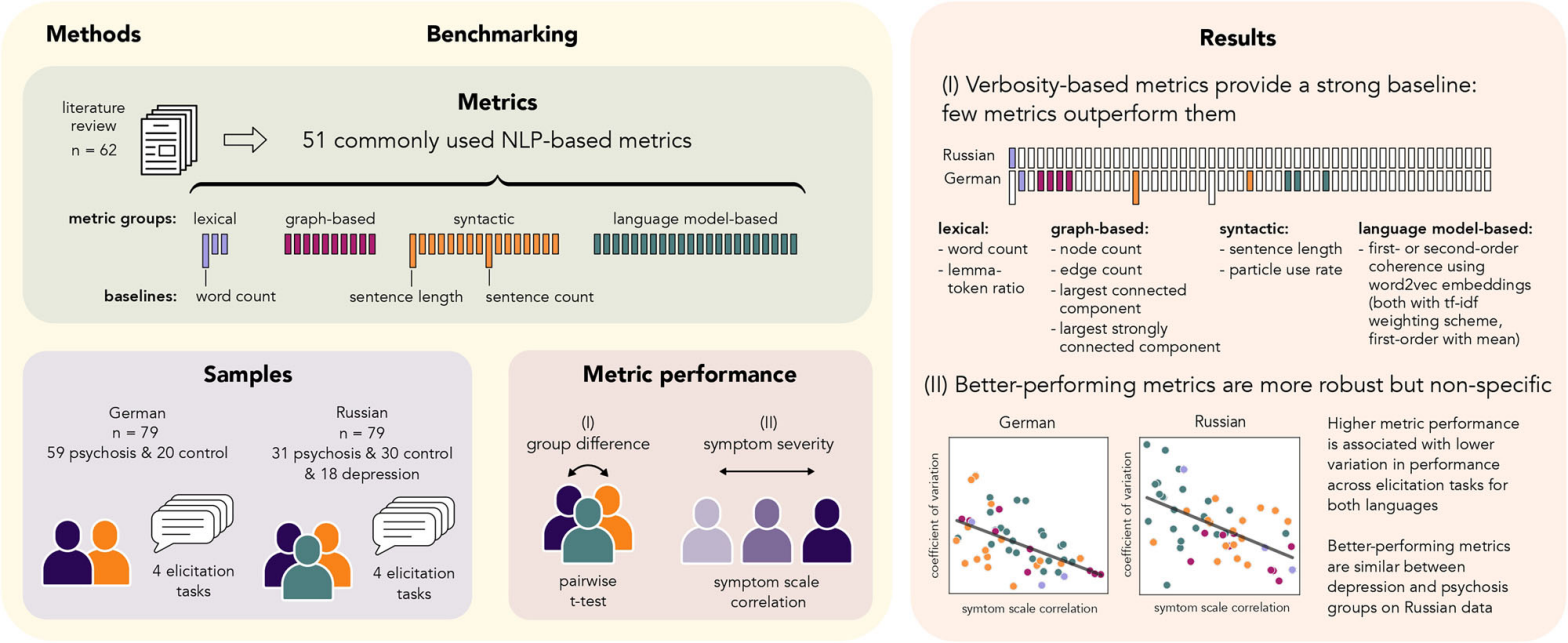
The study framework for benchmarking the commonly used NLP-based psychosis detection methods and an overview of the main findings. **Methods**. ***Samples*** Two datasets of speech samples were used in this study, one with German and one with Russian-speaking participants. Each sample included a group of participants with psychosis as well as a healthy control group and, for the Russian data, a clinical control group of patients with affective disorders with predominantly depressive symptoms. Four different elicitation tasks were used to obtain four texts per participant. ***Metric performance*** Two definitions of metric performance for psychosis detection were used, (i) group difference detection measured with a t-test and (ii) symptom severity assessment measured as correlation with symptom scale scores. ***Benchmarking*** 51 commonly used NLP-based metrics were identified through a literature review comprehending 62 papers, proceedings, and thesis reports. The metrics were grouped into lexical, graph-based, syntactic, and language-model-based. Three verbosity-based baselines were selected, namely, word count, sentence count, and mean sentence length. **Results** The benchmarking identified that only few metrics could outperform the strongest verbosity baseline for symptom severity assessment: graph-based metrics, particle use rate, lemma-token ratio and word2vec first-/second-order coherence for the German sample; no metrics outperformed the strongest baseline for the Russian sample (I). Furthermore, better-performing metrics were more consistent across different elicitation tasks (II).

In this study, we carried out a benchmarking of 51 commonly used NLP-based psychosis detection metrics (Fig. 1**Methods**), selected based on a preliminary literature review (Supplementary Table 1 to 10). The metrics were compared in terms of their ability to assess symptom severity and differentiate between healthy controls and participants with psychosis (Fig. 1**Metric performance**). The benchmarking was carried out on German and Russian language data across four different elicitation tasks (Fig. 1**Samples**). The metrics were compared against three verbosity baselines (Fig. 1**Benchmarking**). The metrics showing the highest performance are listed in Supplementary Table 11. The study found that few metrics could outperform the strongest baseline and that the better-performing metrics were more robust in their performance across elicitation tasks in terms of assessing symptom severity (Fig. 1**Results**). These findings are discussed in detail below.

### Verbosity as a strong baseline

**Fig. 2:**
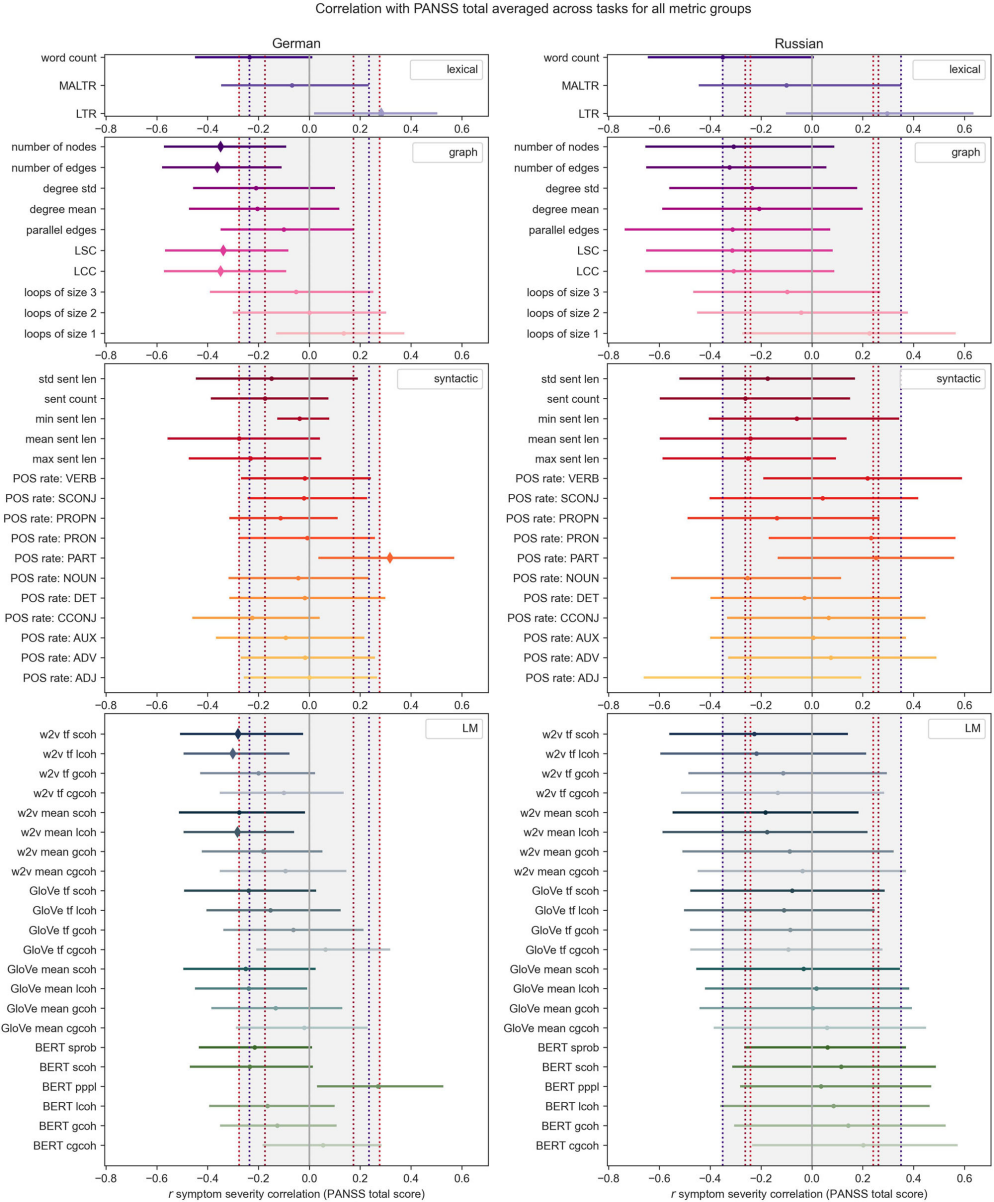
Pearson’s r correlation coefficient with the total PANSS score for each of the tested metrics on the German and Russian datasets averaged across tasks. Each group of metrics is shown as a separate subplot. The word count, mean sentence length, and sentence count baselines are shown with dashed lines, mirrored to represent an absolute value baseline. The gray area indicates a correlation that is weaker than that of the strongest baseline. The intervals around each average indicate 95% confidence intervals obtained with bootstrapping. Each metric is assigned a separate color to facilitate comparison between the two languages. Metrics outperforming the strongest baseline for each language are highlighted with diamond shapes. These were, on the German data, the numbers of nodes and edges, largest connected and strongly connected component sizes, lemma-token ratio, particle use rate, and first- and second-order coherence computed with word2vec (both with TF-IDF weighting, and, for the first-order coherence, with mean weighting). None of the metrics outperformed the strongest baseline on the Russian data.

When compared to each other in the strength of correlation with symptom severity, all but few metrics were outperformed by at least one of the verbosity baselines, with most metrics showing generally weak correlations. Figure 2 shows the comparison of the tested metrics’ correlations with the total Positive and Negative Syndrome Scale (PANSS^59^) score averaged across different elicitation tasks for both languages. PANSS total is presented here as an overview scale. The word count, mean sentence length, and sentence count baselines showed different relative performance for the two languages, with mean sentence length being the strongest baseline for the German dataset (r = −0.28), and word count the strongest baseline for the Russian one (r = −0.35), possibly due to the differences in the elicitation tasks used in the two samples (see Supplementary Figs. 1 and 2 for an analysis of verbosity in the two samples). The metrics that outperformed the best baseline for the German data were as follows. Firstly, the graph-based statistics of the number of nodes and edges, representing lexical diversity and connectedness, the size of the largest connected and strongly connected components, representing graph connectivity (average r = −0.31 for the German). Secondly, the rate of particle use, i.e. words such as ‘not’ or ‘to’. Thirdly, the first-order coherence (between adjacent sentences) and second-order coherence (between sentences one sentence apart), both computed using the word2vec language model (both with TF-IDF weighting, and, for the first-order coherence, with mean weighting). Finally, the ratio of unique lemmas (base forms of words) to the total number of word tokens (i.e., lemma-token ratio: another indicator of lexical diversity).

No metric could outperform the strongest verbosity baseline on the Russian data, word count, as it showed a stronger correlation with total PANSS score compared to the best baseline for the German sample. However, some of the metrics outperforming the strongest baseline on the German data, namely, the graph-based metrics, outperformed the sentence count, the second-best baseline on the Russian data (r = −0.26). Apart from these, the number of parallel edges also outperformed this weaker baseline.

Similar results were obtained for other symptom severity assessments, with the strongest metrics performing across different outcome variables, with the exception of positive symptom severity (Supplementary Figs. 3 and 4). For the PANSS positive subscale, the metrics showed a weaker overall correlation on the German data, as the negative symptoms predominated in this sample. On the Russian data, on the other hand, while no metric could outperform the strongest baseline for other PANSS subscales, for PANSS positive, the largest connected and strongly connected component, the number of nodes, edges, and parallel edges, as well as the lemma-token ratio correlated with symptoms severity more than the strongest baseline.

To quantify the incremental value of each metric beyond verbosity, ΔR^2^ was computed between two ordinary least squares models predicting the PANSS total score as the outcome variable. The base model included major covariates (i.e., age, sex, years of education, and, for the German sample, verbal IQ) along with the best verbosity baseline for each language. This model was compared to a model that additionally included a given metric as a predictor. The observed incremental predictive value generally matched the performance of the metric in terms of correlation with PANSS total (Supplementary Fig. 5). The ΔR^2^ did not exceed 10% of explained variance for any of the metrics in either sample.

As for the difference between participants with psychosis and controls, on the German data, the results demonstrated an even stronger performance of the baselines as compared to that on PANSS total, with only maximum and standard deviation of sentence length outperforming the mean sentence length. On the Russian data, no metric could outperform the strongest baseline in differentiating the psychosis group from the control group (Supplementary Fig. 6). A ROC-AUC analysis revealed similar results, with even stronger performance for the verbosity baselines, no metric outperforming them for either sample (Supplementary Fig. 7).

Finally, there was a substantial correlation between average metric performance and their correlation with verbosity baselines, which was strongest for word count and weakest for mean sentence length (Supplementary Fig. 8).

### Robustness of metric performance

**Fig. 3:**
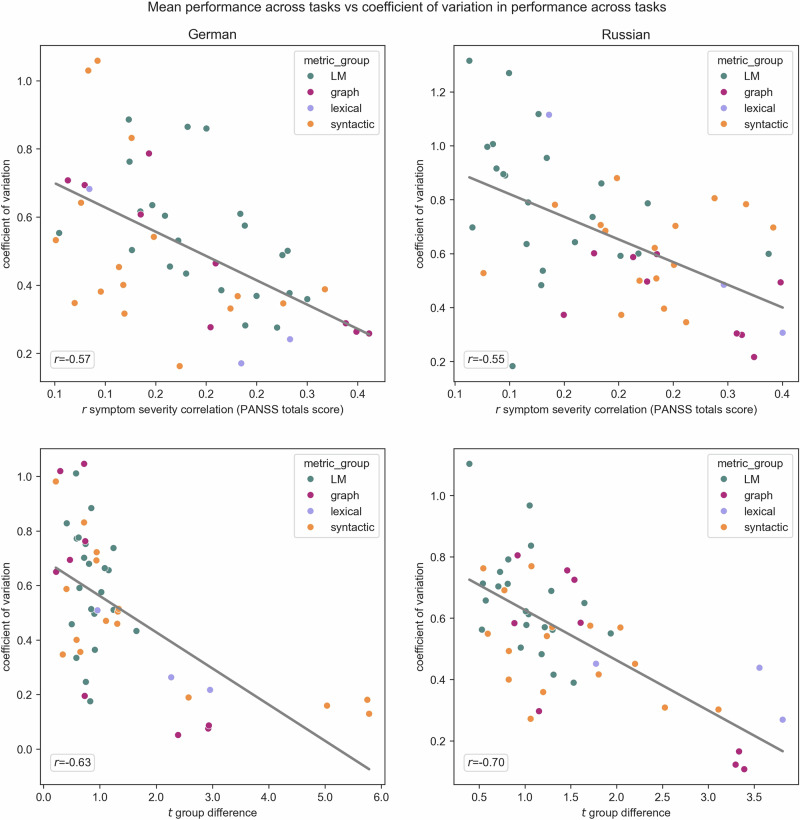
Scatter plot of the coefficient of variation (CV) in metrics’ performance against their mean performance across tasks for correlation with PANSS total score and group difference between individuals with psychosis and controls in both languages. The colors code for the metric group. A linear regression fit is shown with a gray line. Pearson’s r is reported on each panel. Better-performing metrics tended to show less cross-task variance for both symptom correlation and group differentiation.

The analysis of performance consistency showed that metrics which showed higher correlation with symptom severity also tended to show more robust performance across tasks, while weaker metrics were highly task-sensitive. Figure 3 shows the coefficient of variation between the tasks, i.e. how much the results differ between the tasks, plotted against mean performance across all tasks for correlation with symptom severity and group difference for both languages. Although the negative correlation is not particularly strong (*r* between −0.55 and −0.7), this result indicates that the stronger average performance typically comes from better performance on each task, rather than outstanding performance on only one of the tasks. Similar analysis showed that better-performing metrics tended to have more consistency across outcome variables (Supplementary Fig. 9).

Finally, there was more similarity in results between the languages for the well-performing metrics, such as the graph-based ones, as well as the verbosity baselines to some extent. The degree to which these similarities could be analyzed, however, was limited by the fact that the elicitation tasks differed between the languages, since the elicitation task affected the absolute and relative performance of the metrics (ICC2 = 0.58 and 0.37 for PANSS total between-elicitation task agreement on German and Russian, respectively).

### Specificity of metric performance

**Fig. 4:**
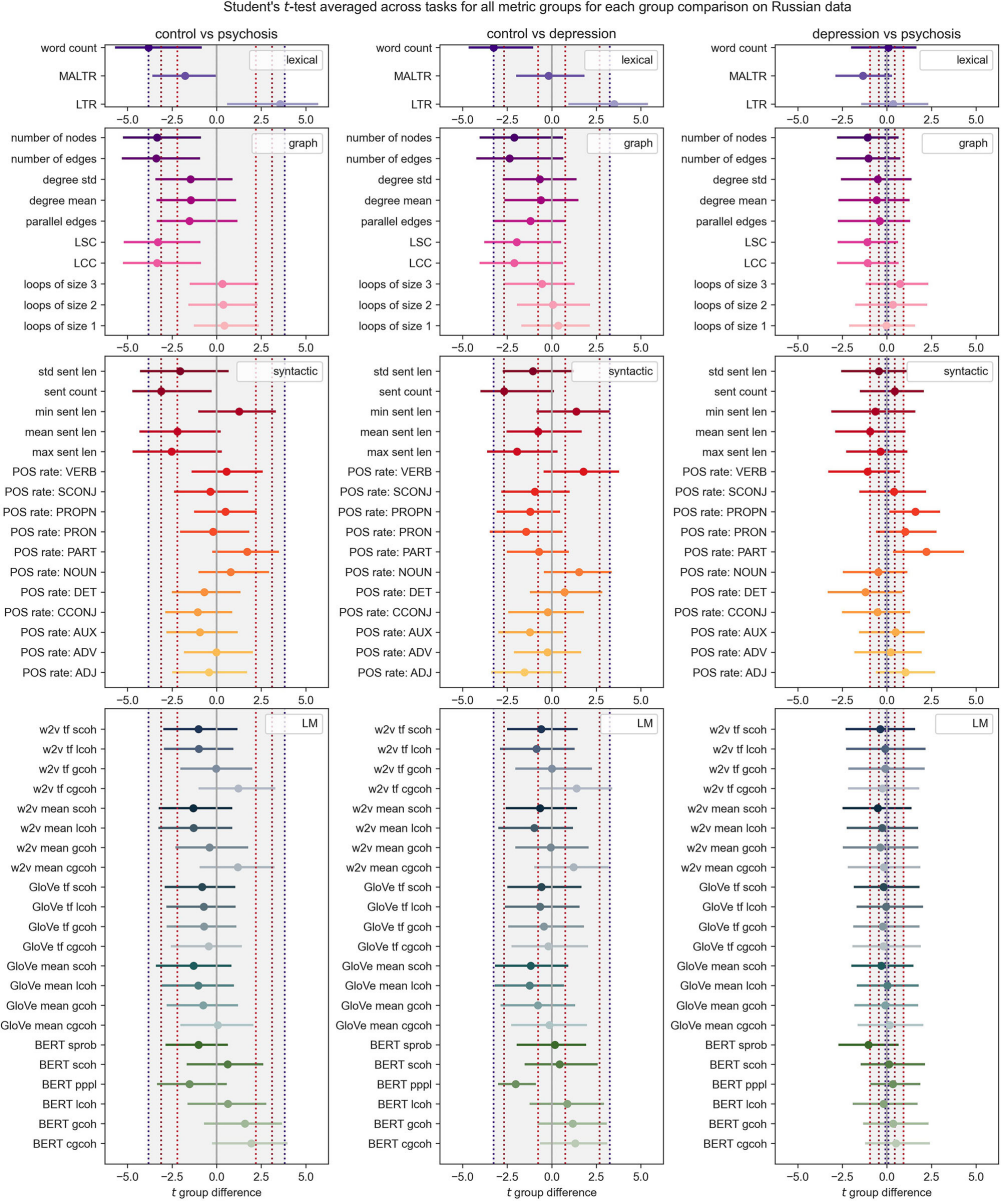
t-test results on the Russian dataset averaged across tasks for control vs psychosis, control vs depression, and depression vs psychosis comparisons. Each group of metrics is shown as a separate subplot. The word count, mean sentence length, and sentence count baselines are shown with dashed lines, mirrored to represent an absolute value baseline. The gray area indicates a correlation that is weaker than that of the strongest baseline. The intervals around each average indicate 95% confidence intervals obtained with bootstrapping. Each metric is assigned a separate color to facilitate comparison between the two languages.

Having observed that the strongest metrics perform more consistently across different tasks and symptom scales, we hypothesized that these metrics might be not specific to psychosis-related speech changes. To investigate that, we performed a t-test comparing the control group and a separate depression group on the Russian data. We then compared the results to those observed for psychosis. This analysis, alongside a t-test comparing the metric values between psychosis and depression groups, is presented in Fig. 4. Metrics that differed most between controls and either of the patient groups overlapped substantially, with the top predictors being similar for both. These included lemma-token ratio, word count, sentence count, number of nodes and edges, as well as the sizes of largest connected and strongly connected components. The t-test results for the difference between control and the two patient groups showed a significant correlation (r = 0.85, p < 0.0001).

Considering the presence of both positive and negative symptoms in the psychosis group, this result suggests that the strongest metrics might capture non-specific symptom severity or linguistic characteristics shared between individuals with depression and psychosis, rather than changes in speech that are only typical for psychosis or schizophrenia. An interesting exception to this pattern was the rate of particle use. This metric differed most between the psychosis and depression groups, and was increased in comparison to control for the psychosis group, but somewhat decreased for the depression one. We believe it is possible that similar results would hold for German if we had depression data available, since all the abovementioned top-performing metrics, excepting sentence count, were among the top-performing ones on the German data as well.

## Discussion

The rise of advanced NLP-based methods in psychosis detection necessitates thorough evaluation and comparison of the proposed metrics^52,53^. In such benchmarking studies, selecting a simple interpretable baseline is of utmost importance as it provides a common reference for all the compared approaches. In our study, we used verbosity metrics, i.e. word count, sentence length, and sentence count, as baselines due to their established use in psychiatric linguistics and because changes in verbosity are an important sign of changes in mental state^37^. In line with previous findings^18,37^, we observed a correlation of verbosity metrics with overall symptom severity on both German and Russian datasets.

Our results demonstrate that among the widely used NLP-based psychosis detection metrics, only a few are able to outperform the verbosity metrics in terms of correlations with symptom severity and between-group differences. Moreover, we observed that higher metric performance is associated with an increased correlation with verbosity. In connection with this result, it is important to note that there is an inherent association between some of the metrics and verbosity. For instance, many lexical metrics are indirectly based on word count, and the same is true of graph-based metrics, though the moving window procedure helps mitigate this dependence^60^. It is also probable that some syntactic metrics, such as part-of-speech rates, are sensitive to overall sentence length; for instance, short sentences are less likely to include coordinating conjunctions. Similar dependencies may affect LM-based metrics like cosine similarity, surprisal, and perplexity^18,29,30,61^. Therefore, this inherent dependence might partially underlie the performance of such metrics. Despite all these considerations, there is no established practice of using verbosity as a baseline in NLP-based psychosis detection. Based on our results, we suggest assessing other NLP markers of symptom severity for potential dependence on verbosity and evaluating advanced models and metrics against verbosity baselines.

In addition to outperforming verbosity baselines, a good NLP metric should perform consistently across different speech elicitation tasks, should be transferable across studies and settings, and, preferably, should perform well for different languages. In our study, better-performing metrics tended to perform more consistently. In the performance of weaker metrics, however, we observed a high degree of variation across tasks and languages. As the tasks differed between the two languages, we cannot differentiate between cross-linguistic and cross-task robustness. It should be noted that the tasks did not only differ in procedure but in emotional content. German participants were explicitly asked to share emotional, personal memories, while tasks in Russian were more neutral. The high variability across tasks which we observed is in line with previous research^29^. In our study, even slight differences between tasks, such as talking about different emotions in the NET interview in the German dataset, yielded different results. It is likely that different elicitation tasks highlight different symptoms. Some of the target symptoms may be easier or harder to detect with any metric depending on the task used. We hypothesize that performance would be more robust on restricted speech tasks, such as the verbal fluency task or single word association task^14,62^, where complex metrics might capture differences that are missed by the most simple baselines. In addition, such tasks may potentially translate more consistently across languages.

One potential cause for the low overall performance and consistency of most metrics may lie in symptom heterogeneity in psychosis^63^, with diverse manifestations of disordered speech and cognitive abilities^64,65^. This is supported by the fact that the best-performing metrics in our study showed the least symptom type specificity, performing similarly across outcome variables. The reason may be that only the simplest linguistic features such as verbosity are shared in the language of all individuals with psychosis. These features could be seen as sensitive to either very common psychotic symptoms or to non-specific symptoms, a general psychiatric distress. The strong correlation between the metric performance for psychosis and depression groups in terms of differentiating patients from controls on the Russian dataset could be interpreted as support for either explanation. The similar performance may reflect a general symptom burden in both conditions or shared characteristics in the speech of individuals with depressive or negative psychosis symptoms, such as poverty of speech and speech content. Indeed, some of the observed differences, including lowered overall verbosity, had already been reported as linguistic markers not only for psychosis but also for depression^66,67^. However, we believe that complex metrics could still be useful for examining some specific speech-related symptoms, such as derailment, in otherwise heterogeneous samples. Longitudinal designs may also elucidate the patterns of metric performance by comparing individuals to their own linguistic baselines, removing interindividual variability, and possibly offering more clinically relevant insights.

Our study has several limitations which primarily concern our clinical samples. First, although comparable in size to others in the field (see Methods), both samples are relatively small. The dataset size and the large number of metrics analyzed precluded meaningful assessment of statistical significance. Sensitivity analyses confirmed that, after Bonferroni correction for 51 comparisons, our samples were only adequately powered to detect very large group differences (Cohen’s d ≥ 1.05 for both the German and Russian dataset), and the study should therefore be interpreted as exploratory. Additionally, combining multiple linguistic markers may yield stronger predictive performance than any single metric evaluated in isolation^34,50,68^; however, our sample size – typical of psychiatric research – precludes reliable multivariate modeling, as overfitting and suppressor effects can inflate apparent predictor contributions in underpowered regression models^69–71^. These considerations also apply to the reported ΔR² values, which may therefore overestimate the true incremental contribution of individual metrics beyond verbosity. Then, the elicitation tasks differed between the two languages, limiting direct cross-linguistic comparisons – future studies should utilize tasks that are comparable in procedure and emotional content when analyzing NLP metrics across different languages. Moreover, it cannot be excluded that more complex NLP metrics (beyond verbosity) would perform better on longer speech samples. As the depression sample was only available for the Russian data, the comparison between depression and psychosis groups could only be carried out for one language. Age and gender distributions differed between samples, though neither correlated with metrics or outcome variables. Then, the predominance of negative symptoms in the German dataset could cause the performance of metrics potentially associated with positive symptoms to be underestimated. Moreover, higher prevalence of negative symptomatology characterised both samples, which might imply that the results for the symptom severity analysis are driven primarily by the PANSS negative subscale score. Lastly, we acknowledge that while identifying the best-performing metrics is important, this is not sufficient to demonstrate true clinical utility. Future research should use our outlined approach and build on the results but also determine which metrics are useful in real-world clinical settings and can improve the lives of people with psychosis^13^. This is especially important considering the risks associated with erroneous predictions of psychiatric symptoms and diagnoses^72^.

We highlight several requirements for future benchmarking studies. First, transcription and preprocessing pipelines should be harmonized across datasets to ensure that differences in metric performance reflect linguistic rather than methodological variance^29^. Second, it should include multiple speech tasks matched across languages in both procedure and emotional content to enable direct cross-linguistic comparisons. Third, more restricted tasks may introduce less variance in benchmarking studies than more open speech elicitation tasks. Finally, verbosity metrics should be included as baselines for other metrics, which should be tested for their dependence on verbosity.

In conclusion, our findings provide a critical foundation for identifying robust, generalizable NLP metrics in psychosis research. Crucially, we demonstrate that simple metrics like verbosity, which might be non-specific markers of psychiatric distress, consistently outperform most other metrics across samples in different languages and speech elicitation tasks. This finding aligns with broader evidence from medical research demonstrating the superiority of simpler models and analyses^73–75^, while the process of creating complexity introduces variance^76^. As the field advances, our approach offers a concrete starting point for validating NLP metrics that are not only scientifically sound but also ready for real-world implementation.

## Methods

### Study approach

The present paper is a benchmarking study, meaning that it assesses the performance of many common approaches used in psychosis detection, comparing them against each other and against several pre-selected baseline approaches serving as a common reference point. All the selected metrics are evaluated on three distinct dimensions: (i) their ability to differentiate between healthy controls and participants with psychosis, (ii) their correlation with symptom severity, and (iii) the robustness of their performance across different speech elicitation tasks, languages, and outcome variables. These dimensions are treated as separate and performance on one of them does not imply performance on the others.

### Metric selection

To select the most frequently used metrics for our benchmarking, we conducted a literature review of 62 papers, theses, and conference proceedings. The papers were identified by keywords relating to psychotic disorders and NLP, and the search was expanded with the cited works. Over 125 metrics were screened, and 51 were selected for this study based on the frequency of use, reported performance, and cross-linguistic / cross-study applicability. As mentioned above, the literature review is summarized in Supplementary Table 1 to 10.

The metrics were grouped based on the linguistic units they operate over into lexical, syntactic, and semantic, with the latter separated further into language-model-based and graph-based metrics. The **lexical** metrics included word count, lemma-token ratio (i.e., the ratio of unique base forms of words to total words), and moving average lemma-token ratio. The **syntactic** metrics included the mean, minimal, and maximal sentence length and sentence count, as well as the relative parts-of-speech frequency (i.e., the proportion of nouns, verbs, adjectives, etc. relative to total word count). The **graph-based** metrics were calculated using lemma co-occurrence over a moving window of 100 words and included the node and edge counts, (where nodes are lemmas, and edges are the connections between them), sizes of the largest connected and strongly connected components, the mean and standard deviation of the node degree, the number of parallel edges, and the number of loops of length one (repeated words), two, and three. The **language-model-based** metrics were calculated using the most frequently used models, namely, word2vec (w2v)^77^, GloVe^78^, and BERT^79^, and included first- and second-order coherence, global and cumulative global coherence for each model, as well as next-sentence probability and pseudo-perplexity obtained from BERT. The word2vec and GloVe vectors were aggregated into sentence vectors using word average and TF-IDF weighting schemes. Formulae for the embedding-based LM-based metrics are listed in Table 1.

**Table 1:** Formulae for the embedding-based metrics

Metric Name	Definition
First-Order Coherence (local coherence)	${ag}{{g}_{i=1}}^{N}({cossim}({S}_{i},\,{S}_{i+1}))$
Second-Order Coherence	${ag}{{g}_{i=1}}^{N-1}({cossim}({S}_{i},\,{S}_{i+2}))$
Centroid Global Coherence	${ag}{{g}_{i=1}}^{N}({cossim}({S}_{i},{mea}{{n}_{j=1}}^{N}({S}_{j})))$
Cumulative Centroid Global Coherence	${ag}{{g}_{i=1}}^{N}({cossim}({S}_{i},{mea}{{n}_{j=1}}^{i}({S}_{j})))$

S - sentence embedding; ${cossim}$ - cosine similarity; ${agg}$ - aggregation scheme (such as mean, max, min, or median); N - number of sentences.

As changes in verbosity commonly accompany psychotic disorders^18,37^, three verbosity-capturing metrics were selected to be used as potential baselines: word count, mean sentence length, and sentence count. Total word count can be seen as an aggregate metric which is comprised of sentence length and count.

### Datasets

The data used in the present study included a German- and a Russian-speaking sample. The German sample included 59 individuals with a diagnosis of schizophrenia or schizoaffective disorder (according to DSM-IV-TR, confirmed by trained clinicians) and 20 controls. PANSS was used to assess the symptom severity among individuals with psychosis, with negative symptoms predominating in the sample. Additionally, verbal IQ was assessed for each patient^80^. A short version of the Narrative of Emotions Task^81^ interview was used as the elicitation task. The interview included three open-ended questions on four emotions: sadness, fear, anger, and happiness, which were processed as separate parts of the interview. There was no difference in age, education years, or clinical scale scores between the sexes in the clinical sample. There was also no correlation with years of education or verbal IQ in any of the symptom severity scales, while the symptom severity scales were intercorrelated.

The Russian sample consisted of 31 individuals with a diagnosis of schizophrenia or schizoaffective disorder (according to ICD-10, confirmed by a trained clinician), 30 controls, and a clinical control group of 18 individuals with various affective disorders (including depressive disorders and bipolar disorder) with predominantly depressive symptoms, measured using Hamilton Depression Rating Scale^82^. PANSS scores were collected for all participants. Four different elicitation tasks were used: two picture-elicited tasks (‘sportsman’ and ‘adventure’), one instruction task (‘chair’) and one personal story task (‘present’). The picture description tasks were elicited using two Bidstrup comics, asking the participant to tell the story depicted in them. The instruction task was elicited using an IKEA chair brochure, asking the participant to instruct a third person, who cannot see the image. Finally, the personal story task was elicited by asking the participant to tell a story about the most memorable present that they had received. Each task was analyzed separately. There were no differences in age between individuals with psychosis or depressive symptoms and controls, but the difference in the years of education was significant (t = 3.6, p = 0.0006). There were no differences between the sexes in either sample. After Bonferroni correction, only one of the clinical rating scores correlated significantly with years of education, namely, PANSS general (r = −0.41, p before correction = 0.0008). The psychiatric scores were also intercorrelated. The characteristics of the two datasets are summarized in Table 2.

**Table 2:** Characteristics of the two datasets

Language	Group	n	Female	Age	Years of education	PANSS total score	Word count
German	psychosis	59	24	39.5 (11.1)	14.6 (3.0)	57.3 (16.2)	144.72 (107.59)
	healthy controls	20	9	43.85 (13.3)	15.5 (2.8)	-	231.82 (133.14)
Russian	psychosis	31	25	27.13 (7.14)	13.32 (2.41)	69.79 (16.13)	92.74 (62.87)
	depression	18	18	20.89 (3.71)	12.67 (1.94)	37.92 (5.89)	95.64 (38.37)
	healthy controls	30	26	25.0 (7.4)	15.43 (2.13)	30.77 (1.54)	184.25 (97.17)

Standard deviation is provided in parenthesis for each mean value. Word count = total number of spoken words averaged per task. “depression” refers to the sample of patients with affective disorders with predominantly depressive symptoms.

The psychiatric symptom scale values are presented in Table 3. In both datasets, the negative symptoms were the most prevalent as measured by the PANSS negative subscale.

**Table 3:** Clinical statistics of the two samples

language	group	verbal IQ	PANSS total score	PANSS negative score	PANSS positive score	PANSS general psychopathology score	SANS score	SAPS score
	range		30-210	7–49	7–49	16–112	0–120	0–170
German	psychosis	105.2 (15.7)	57.3 (16.2)	16.9 (6.0)	12.7 (5.5)	27.8 (7.5)	27.7 (20.4)	16.8 (16.7)
	control							
Russian	psychosis		69.79 (16.13)	22.93 (8.59)	15.90 (4.92)	30.97 (8.42)		
	depression		37.92 (5.89)	8.31 (1.97)	8.46 (1.94)	21.15 (3.58)		
	control		30.77 (1.54)	7.23 (0.53)	7.23 (0.61)	16.32 (0.95)		

Standard deviation is provided in parenthesis for each mean value. The range is provided for the possible values of the psychiatric scales. “depression” refers to the sample of patients with affective disorders with predominantly depressive symptoms.

Both samples were analyzed to check for the effects of the control variables, namely, age, verbal IQ, education years, and sex, on the outcome variables, i.e. psychiatric scales and group difference, as well as on the metrics compared in the study.

After correcting for multiple testing, there was no relation between sentence length and sex, age, years of education, or verbal IQ in the two datasets. In the German sample, there was a significant negative correlation of symptom severity with mean sentence length for the PANSS negative score (r < −0.4, p before correction < 0.001).

As for the relation between the control variables and the tested metrics, after Bonferroni correction, there was no difference in metric between men and women, and no significant correlation with age, years of education, or verbal IQ, though this lack of significance may be due to the high number of comparisons.

### Preprocessing

Each text was manually transcribed, with the interviewer’s speech, as well as filled hesitation pauses removed from the transcripts. The sentence segmentation procedure was the same in both datasets and followed the guidelines described in previous work^19^. The texts were then automatically tokenised and lemmatized. The lemmatization and part-of-speech-tagging were performed using the SpaCy10 de_core_news_md and ru_core_news_md models.

### Analysis

We used bootstrapping (1000 samples) to account for the effect of influential points and to assess metric stability with respect to them. Three dimensions of performance were evaluated independently on each bootstrap sample and aggregated across them. First, case-control discrimination was assessed using a two-sided independent samples t-test comparing individuals with psychosis and healthy controls, and, for the Russian sample, between each of these groups and the depression group. An additional ROC-AUC analysis was performed for a logistic regression with individual metrics as predictors for each group comparison on both languages. Second, Pearson’s r was used for assessing the correlation with the symptom severity scales. Third, robustness was defined as consistency of each metric’s performance across these two dimensions over different speech elicitation tasks and languages. Pearson’s r was used to assess the correlation between the metrics and each of the verbosity baselines. Finally, ΔR^2^ between ordinary least square regressions onto PANSS total was used to evaluate the incremental value of each metric over the best verbosity baseline for each language. The baseline model included the major covariates (age, sex, years of education, and, for the German sample, verbal IQ) alongside the best verbosity metric, and this model’s R^2^ was compared to that of a model that included one additional metric. All analyses of symptom severity were performed on the psychosis group only.

The present study, as it is a benchmarking, is most concerned with the relative performance of metrics across these dimensions, and not the significance of each test. Moreover, the number of comparisons would have been prohibitively large for any of the results to remain significant after corrections. Therefore, no p-values are reported, with the exception of control variable analysis, which was performed on the data before bootstrapping.

We performed sensitivity analysis, which determined the minimum effect size detectable given our sample sizes and the number of metrics examined. Given that 51 metrics were tested per dataset, we applied a Bonferroni correction. Cohen’s d was calculated for a one-tailed independent-samples t-tests with 80% power.

### Human Ethics and Consent to Participate

All participants in both samples provided written informed consent to take part in the experiment. The study was approved by the local ethics committees (Ethikkommission der Charité – Universitätsmedizin Berlin, approval number: EA1/200/15; and Ethical Committee of the Moscow Federal State Budgetary Scientific Institution “Scientific Center for Mental Health”, approval number 746 granted on 18.03.2021).

## Supplementary information

Supplementary_Information

## Data Availability

The raw, patient-level personally identifiable data (age, sex, severity) and transcripts that support the findings of this study are not available for privacy reasons. The sample averages grouped by diagnostic status as well as the bootstrap correlation values for all metrics are available on GitHub as an excel table (https://github.com/flying-bear/NLP_NAP_Benchmarking). The data supporting this study’s findings are from Charité Universtätsmedizin Berlin and Moscow Mental Health Centre. De-identified data may be accessed from the authors upon reasonable request. For requests from academic researchers, access will be evaluated by the data access committee and a response given within one month. To request data, please contact Dr. Sandra Just at sandra.a.just@uit.no.

## Code availability

The code developed for the processing, analysis, and visualizations in this paper is available in the GitHub repository: https://github.com/flying-bear/NLP_NAP_Benchmarking.
