# Supplementary material for: Cross-linguistic benchmarking of NLP metrics for psychosis research
Source: NPJ Digit Med. 2026 Aug 3;9:594. doi: 10.1038/s41746-026-03053-y (PMC13433947; doi:10.1038/s41746-026-03053-y)
Supplement: Supplementary file 1 [file 41746_2026_3053_MOESM1_ESM.pdf]

S1 Supplementary Figures for the Results Section

1.1. Sentence length vs sentence count

The difference in relative baseline performance between the two datasets may partially be caused by the differences between the elicitation tasks used in them. The elicitation tasks, as shown in the images below, strongly affected the verbosity and its composition, i.e. sentence count vs sentence length as the major factor in the overall word count.

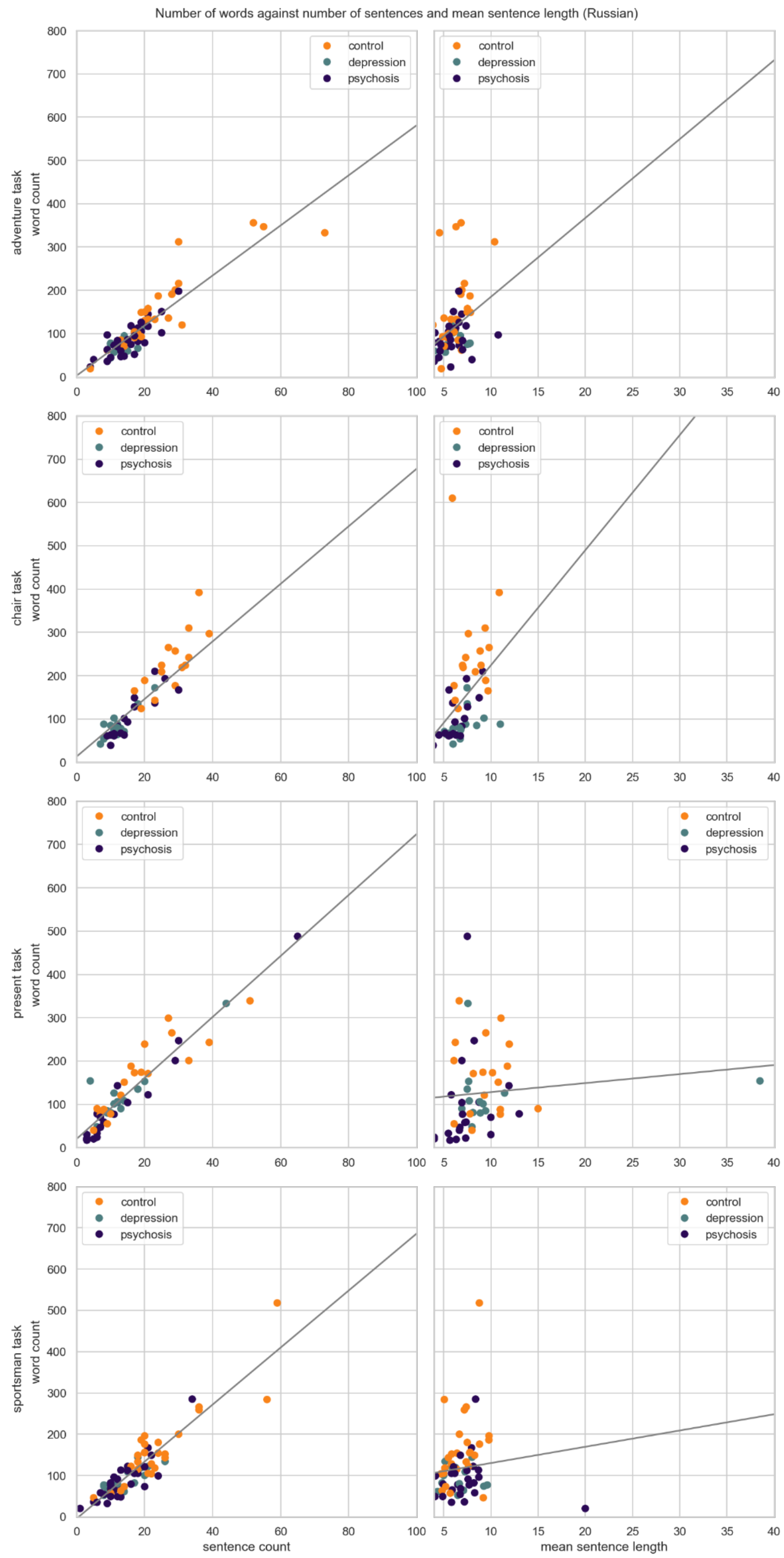

SUPPLEMENTARY FIGURE 1: Scatter plot of the mean sentence length and sentence count against total word count for each task in the Russian sample. Each group is represented with a different color. “control” stands for the sample of the healthy participants. “depression” is the sample of patients with affective disorders with predominantly depressive symptoms. A linear regression fit is shown with a gray line.

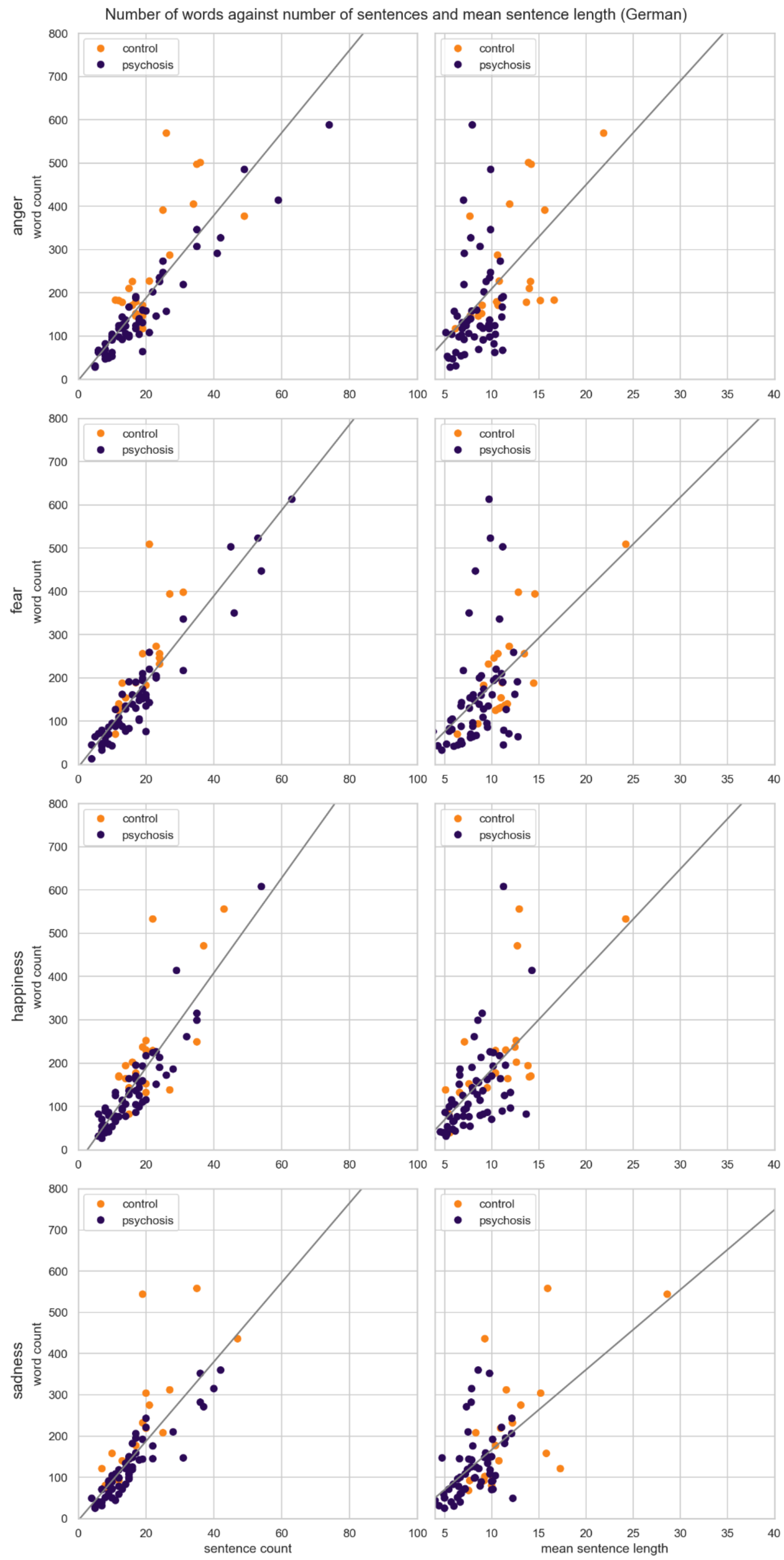

SUPPLEMENTARY FIGURE 2: Scatter plot of the mean sentence length and sentence count against total word count for each task in the German sample. Each group is represented with a different color. “control” stands for the sample of the healthy participants. A linear regression fit is shown with a gray line.

1.2. Correlation with PANSS subscales averaged across tasks

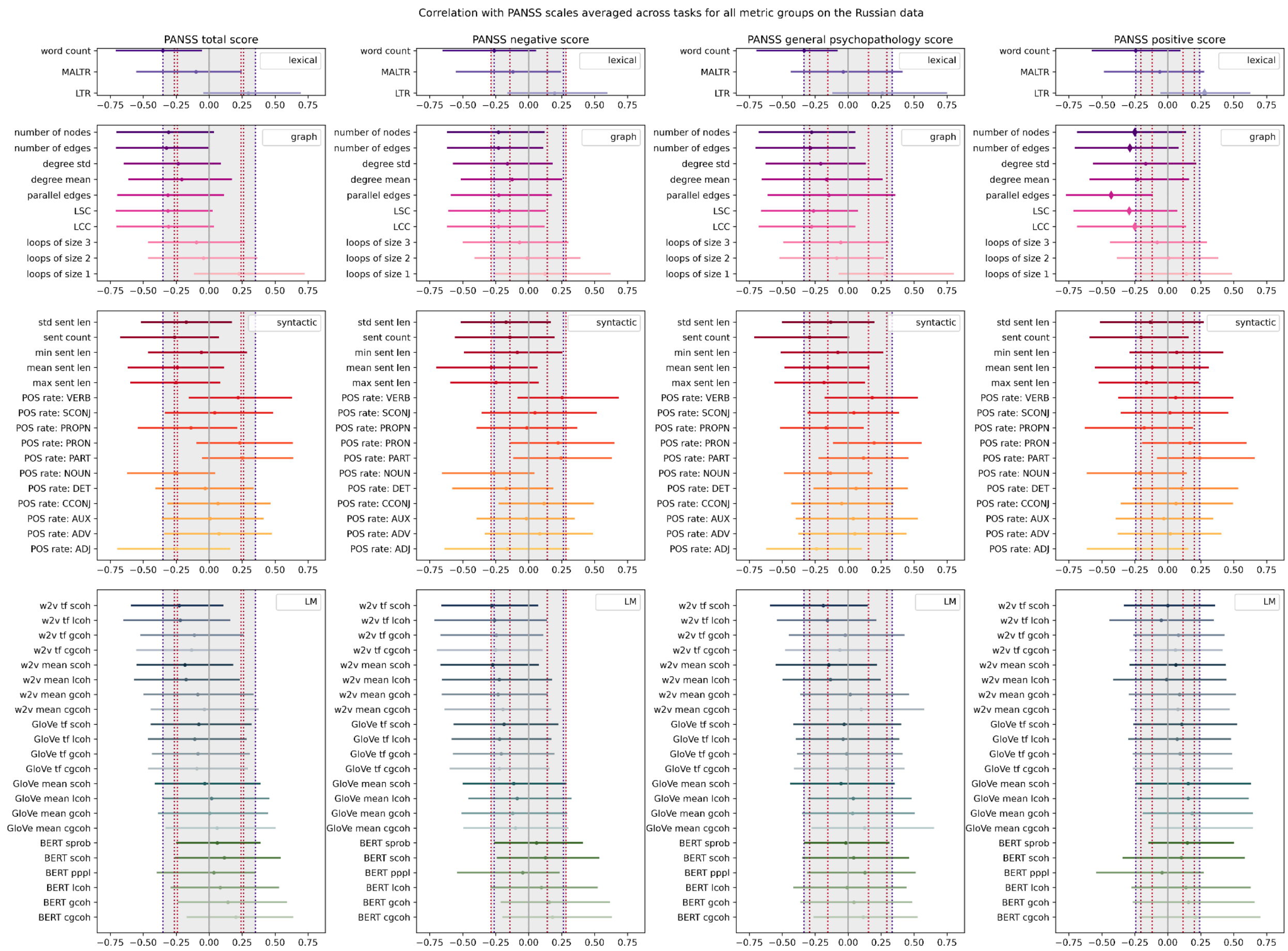

SUPPLEMENTARY FIGURE 3: Pearson's r correlation coefficient with the PANSS subscales for each of the tested metrics on the Russian dataset averaged across tasks. Each group of metrics is shown as a separate subplot. The word count, mean sentence length, and sentence count baselines are shown with dashed lines, mirrored to represent an absolute value baseline. The gray area indicates a correlation that is weaker than that of the strongest baseline. The intervals around each average indicate 95% confidence intervals obtained with bootstrapping. Each metric is assigned a separate color to facilitate comparison between the two languages. Metrics outperforming the strongest baseline for each language are highlighted with diamond shapes.

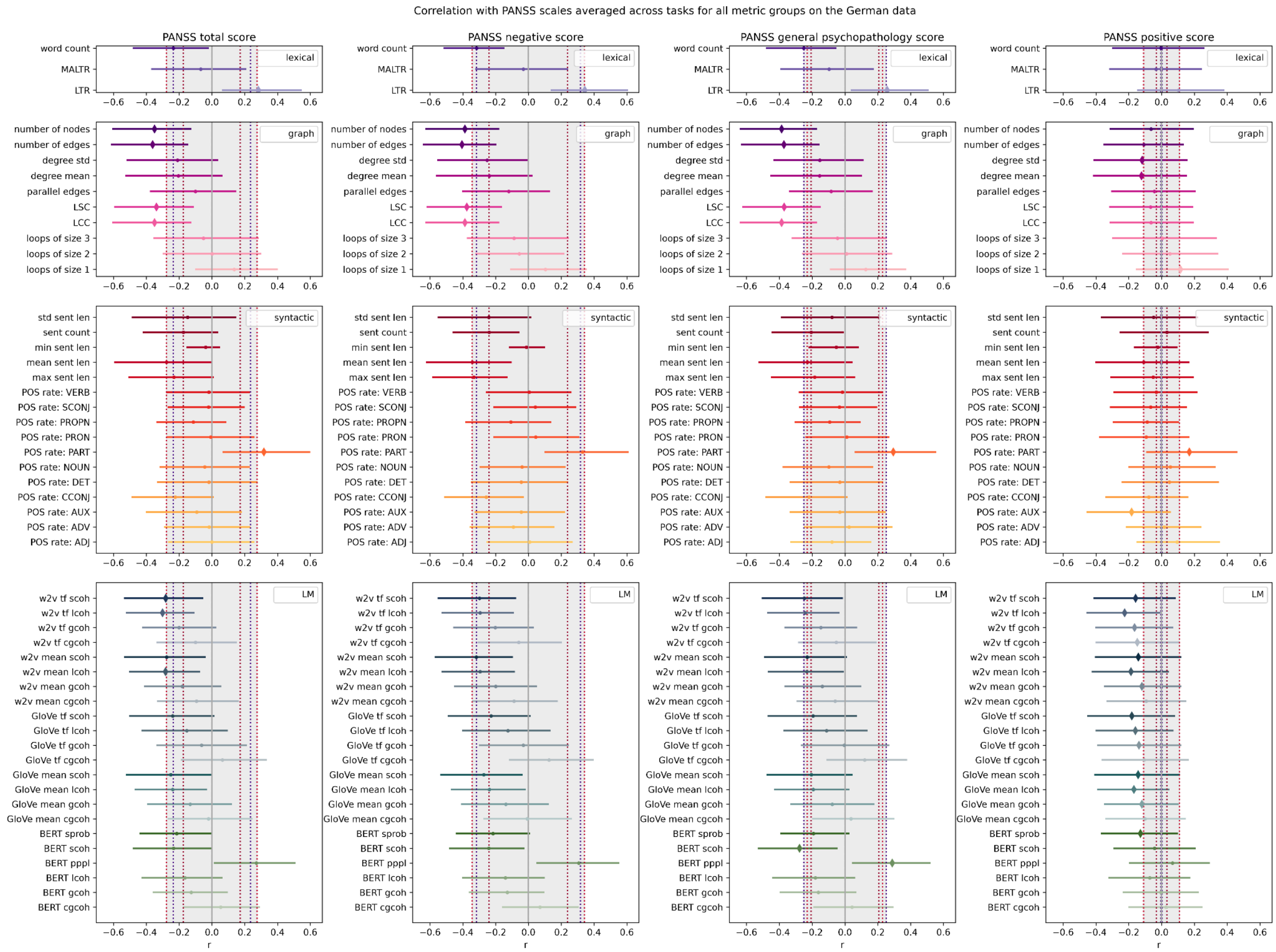

SUPPLEMENTARY FIGURE 4: Pearson's r correlation coefficient with the PANSS subscales for each of the tested metrics on the German dataset averaged across tasks. Each group of metrics is shown as a separate subplot. The word count, mean sentence length, and sentence count baselines are shown with dashed lines, mirrored to represent an absolute value baseline. The gray area indicates a correlation that is weaker than that of the strongest baseline. The intervals around each average indicate 95% confidence intervals obtained with bootstrapping. Each metric is assigned a separate color to facilitate comparison between the two languages. Metrics outperforming the strongest baseline for each language are highlighted with diamond shapes.

1.3. ΔR^2 results for PANSS total averaged across tasks

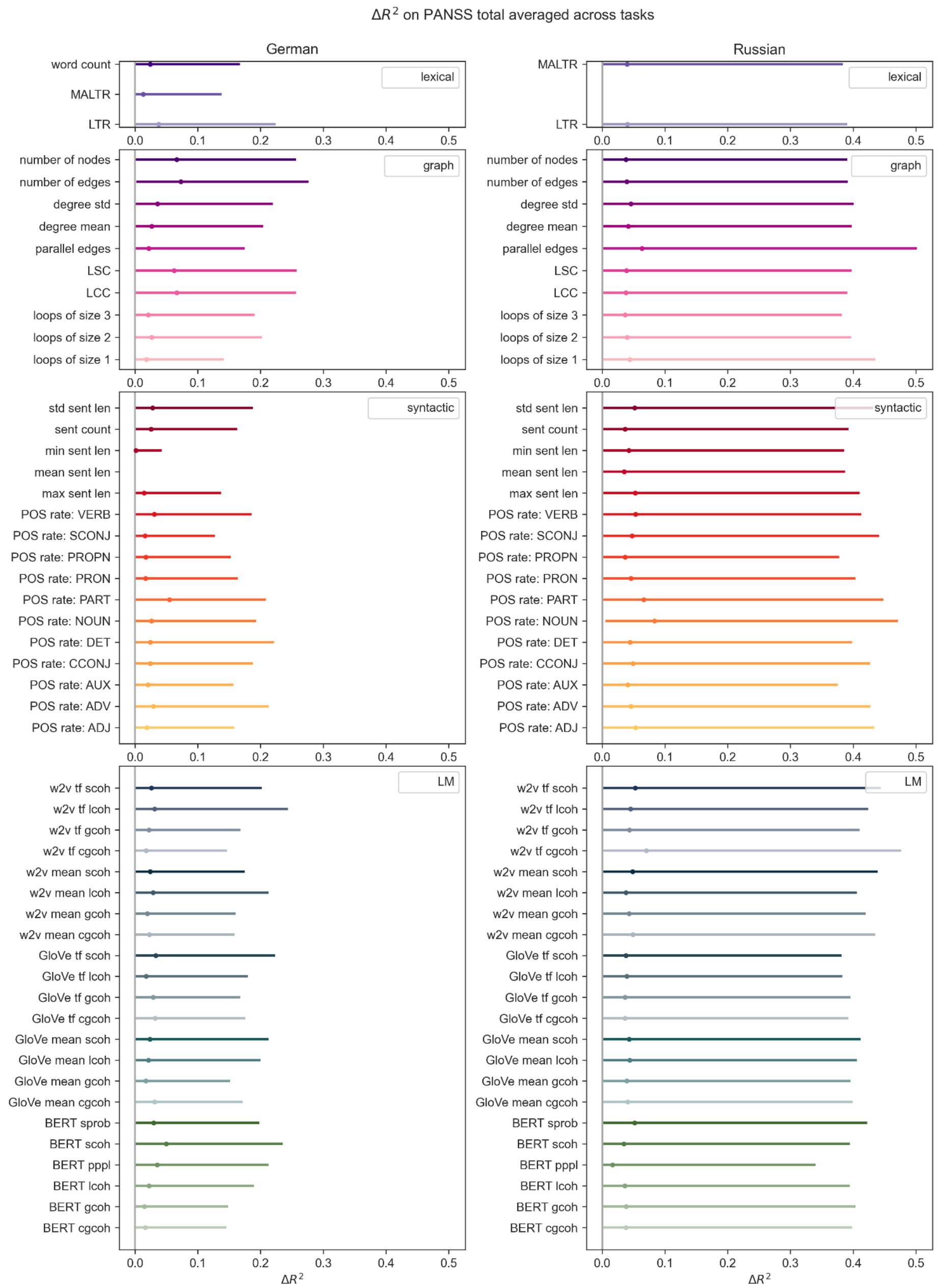

SUPPLEMENTARY FIGURE 5: ΔR^2 coefficient for the PANSS total for each of the tested metrics on the German and Russian datasets averaged across tasks. Each group of metrics is shown as a separate subplot. The intervals around each average indicate 95% confidence intervals obtained with bootstrapping. Each metric is assigned a separate color to facilitate comparison between the two languages. ΔR^2 was computed between two ordinary least squares models predicting the PANSS total score as the outcome variable. The base model included major covariates (i.e., age, sex, years of education, and, for the German sample, verbal IQ) along with the best verbosity baseline for each language. This model was compared to a model that additionally included a given metric as a predictor.

1.4. t-test results averaged across tasks

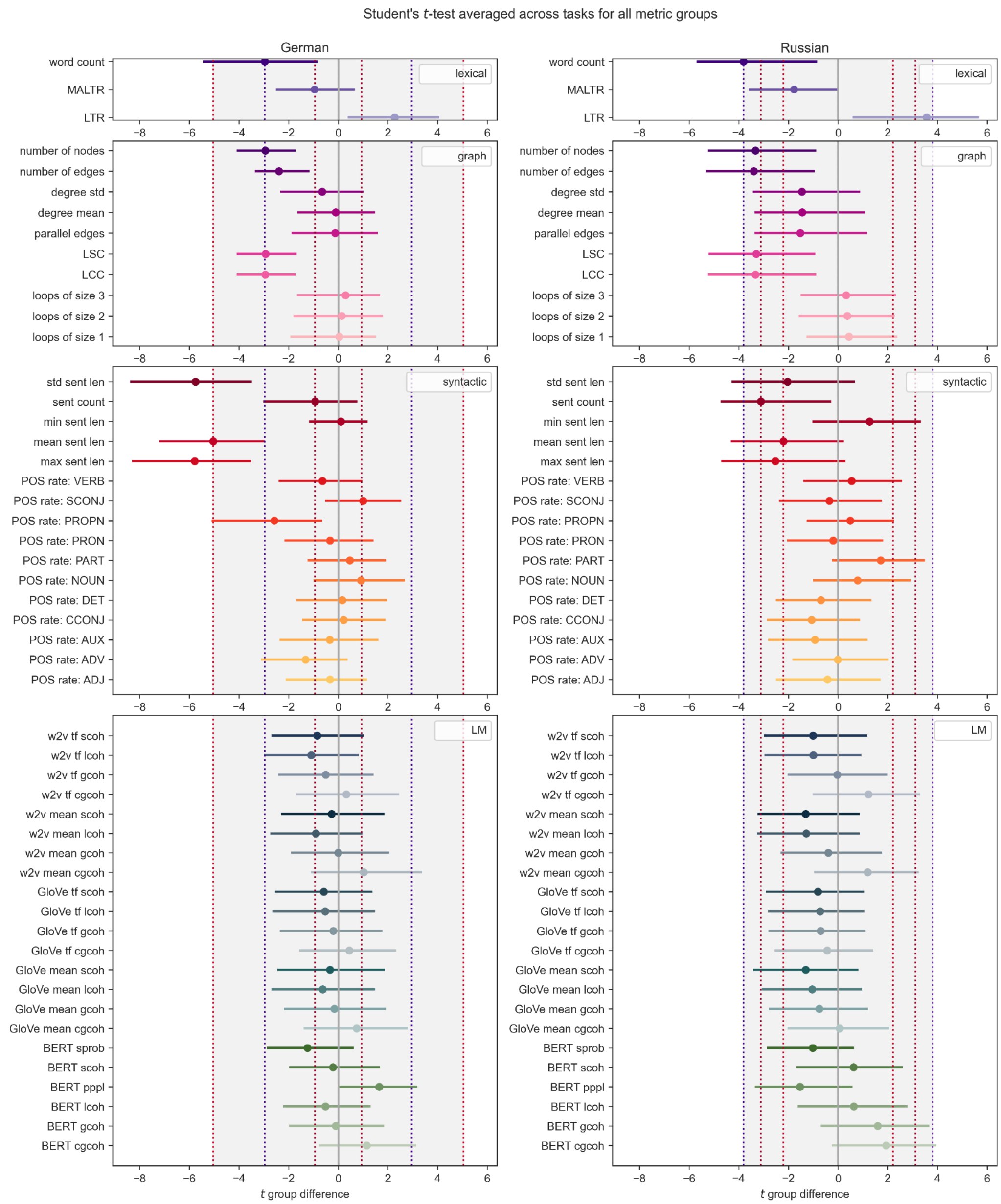

SUPPLEMENTARY FIGURE 6: t-test results on psychosis vs control groups for each of the tested metrics on the German and Russian datasets averaged across tasks. Each group of metrics is shown as a separate subplot. The word count, mean sentence length, and sentence count baselines are shown with dashed lines, mirrored to represent an absolute value baseline. The gray area indicates a correlation that is weaker than that of the strongest baseline. The intervals around each average indicate 95% confidence intervals obtained with bootstrapping. Each metric is assigned a separate color to facilitate comparison between the two languages. Metrics outperforming the strongest baseline are, for German, maximal sentence length and standard deviation in sentence length, both being quite close to the strongest baseline of mean sentence length.

1.5. AUC analysis results averaged across tasks

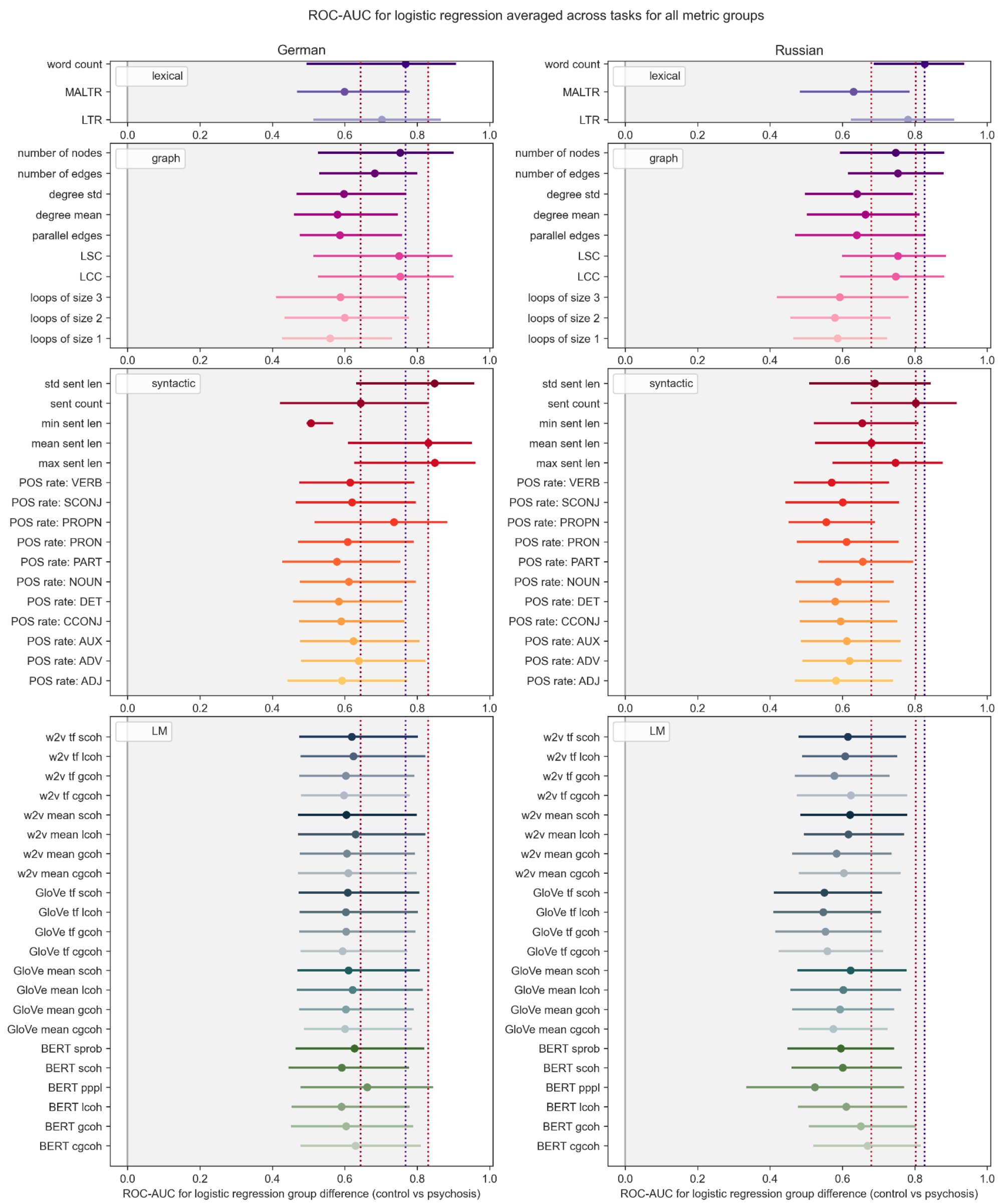

SUPPLEMENTARY FIGURE 7: Area under the receiver operating characteristic curve (ROC-AUC) analusis results for a logistic regression of a single metric onto on psychosis vs control groups for each of the tested metrics on the German and Russian datasets averaged across tasks. Each group of metrics is shown as a separate subplot. The word count, mean sentence length, and sentence count baselines are shown with dashed lines, mirrored to represent an absolute value baseline. The gray area indicates a correlation that is weaker than that of the strongest baseline. The intervals around each average indicate 95% confidence intervals obtained with bootstrapping. Each metric is assigned a separate color to facilitate comparison between the two languages.

1.6. The relation between performance and correlation with verbosity

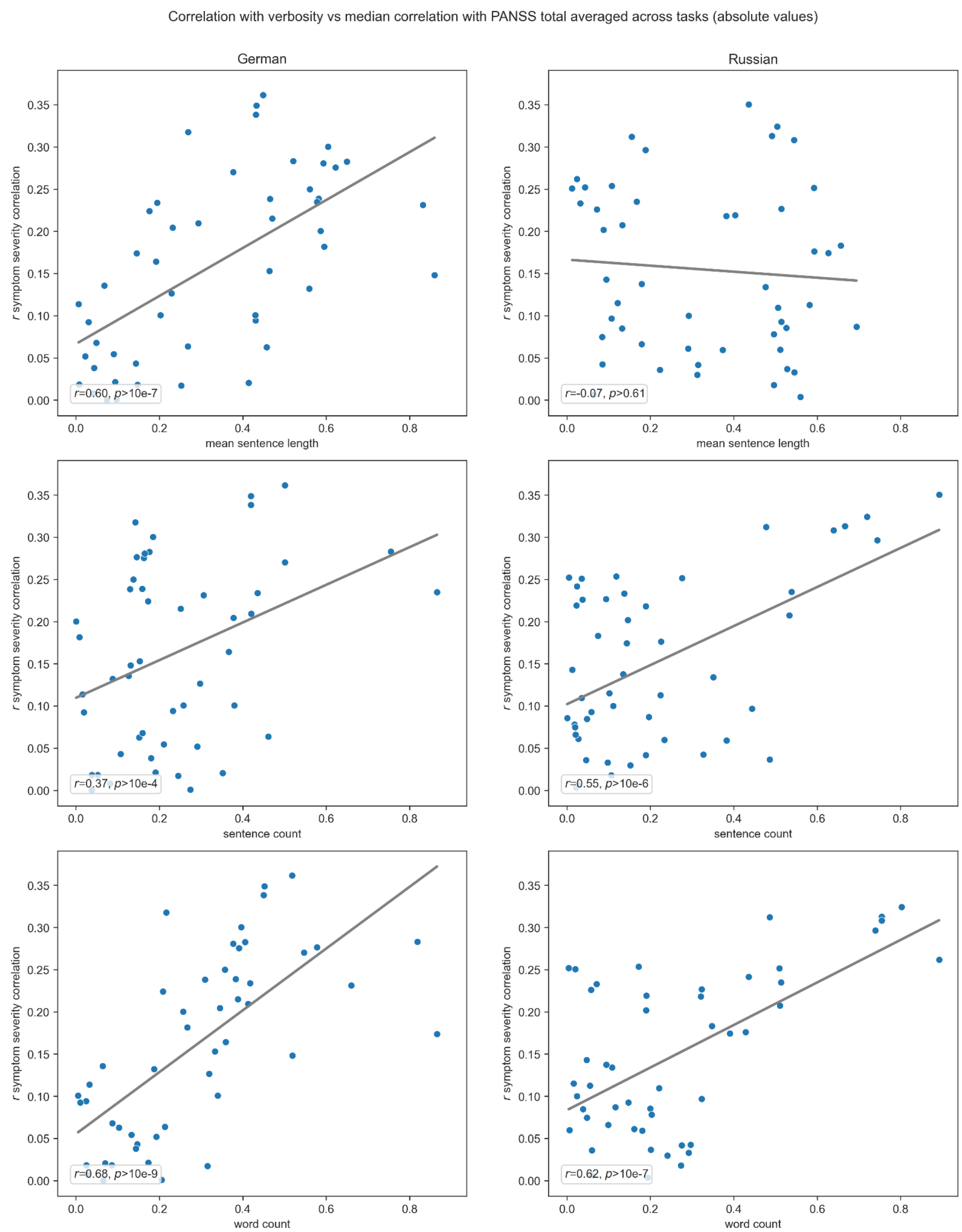

SUPPLEMENTARY FIGURE 8: Scatter plot of metric performance on PANSS total against the correlation with each of the verbosity baselines averaged across tasks. A linear regression fit is shown with a gray line. Pearson's r and the corresponding p-value (uncorrected) are reported on each pane.

1.7. Best metrics perform across scales

To assess how much the metrics performed differently on the different outcome variables, the performance on each scale had to be normalized to the same range using Z-normalization. This was applied to the t-test, PANSS total score, PANSS subscale scores. Then, the coefficient of variance and the mean scores were calculated over the normalized performance values.

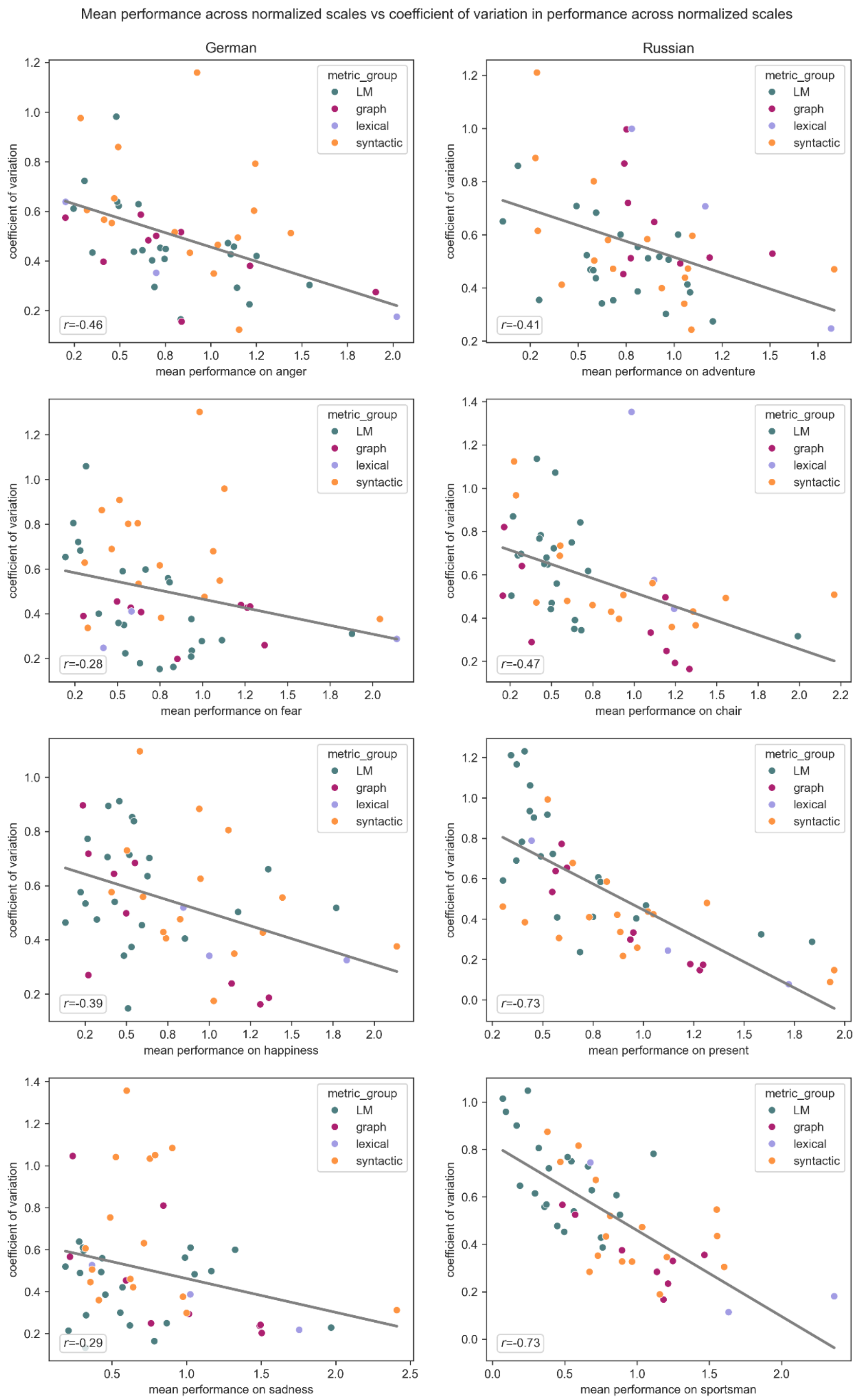

SUPPLEMENTARY FIGURE 9: Scatter plot of the normalized metric performance against the coefficient of variation in performance across scales for each task. The colors code for the metric group. A linear regression fit is shown with a gray line. Pearson's r is reported on each pane.

S2 Supplementary Information on the Literature Review

The literature review was performed at the end of 2023. The keywords used were “psychosis”, “schizophrenia”, and “thought disorder” as well as “automated” + “language”, “embedding”, “model”, and “NLP”. The citations were used to expand the search. Purely manual metrics were excluded from the review.

In our literature review, the mode dataset size was 59 patients and 44 controls, and the mean - 20 patients and 21 controls.

2.1. Lexical metrics

**Lexical metric**	**Count**	**+**	**-**	**!**	**NR**	**Corr. Symptoms**	**No Corr. Symptoms**	**Classifier Feature**
word count	29	Iter et al. 2018[1]	Mota et al. 2014[2]; Willits et al. 2018[3]; Doré 2019[4]; Just et al. 2019[5]; Just et al. 2020[6]; Panicheva & Litvinova 2019[7]; *Morgan et al. 2021*[8]*; Spencer et al. 2021*[9]; Voppel et al. 2021[10]; Liang et al. 2022[11]; Parola et al. 2023[12]; *Nettekoven et al. 2023*[13]	Mota et al. 2012[14]*; Gupta et al. 2018*[15]; Ryazanskaya 2020[16]; *Morgan et al. 2021*[8]*;* Tang et al. 2021[17]; Alonso-Sánchez et al. 2022[18]; Alonso-Sánchez et al. 2023[19]; Schneider et al. 2023[20]	Kořánová 2017[21]; *Argolo et al. 2023*[22]	*Morgan et al. 2021*[8]; Minor et al. 2023[23]		Liebenthal et al. 2023[24]; Tang et al. 2023a[25]; Tang et al. 2023b[26]; Voppel et al. 2023[27]
LD: TTR+	8	Ziv et al. 2022[28]	Willits et al. 2018[3]; Aich et al. 2022[29]; Minor et al. 2023[23]	*Hitczenko et al. 2021*[30]; Schneider et al. 2023[20]			Jeong et al. 2023[31]	Rosenstein et al. 2015[32]; Kramov, 2020[33]; Liang et al. 2022[11]; Tang et al. 2023b[26]
LD: unique words	2		Willits et al. 2018[3]	Schneider et al. 2023[20]				
LD: Honoré statistics	1					Jeong et al. 2023[31]		
SA: emotion words	6	Mitchell et al. 2015[34]	Mitchell et al. 2015[34], Aich et al. 2022[29]	Mota et al. 2023[35]		Vail et al. 2018[36], *Girard et al. 2022*[37]*,* Minor et al. 2023[23]		
SA: models	5			*Argolo et al. 2023*[22]				Sarzynska-Wawer et al. 2021[38]; Tang et al. 2023a[25], Tang et al. 2023b[26]; Aich et al. 2022[29];
neologisms, OOV	2	Just et al. 2019[5], Just et al.						

		2020[6]						
foreign word use	1					Jeong et al. 2023[31]		

SUPPLEMENTARY TABLE 1: Summary of the results reported for the lexical metrics used in the reviewed papers.
"+" indicates a significant group difference with higher values in the patient population. "-" indicates a significant group difference with lower values in the patient population. "!" indicates the absence of significant differences in the metric tested. "NR" indicates that the metric is used but results are not reported. "Corr. Symptoms" indicates a significant correlation with symptom severity in any direction. "Classifier Feature" indicates that the metric is used as a feature in a classifier or latent analysis.
The studies on clinical high-risk populations are shown in italics. * - no correlation with symptoms.
"LD" stands for lexical diversity. "SA" stands for sentiment analysis. "OOV" stands for out-of-vocabulary words.

2.2. Syntactic metrics

**Syntactic metric**	**Count**	**+**	**-**	**!**	**NR**	**Corr. Ling. Scale**	**Corr. Symptoms**	**No Corr. Symptoms**	**Classifier Feature**
POS: det, wh-words	10	Mitchell et al. 2015[34]	*Corcoran et al. 2018*[39]; Sarzynska-Wawer et al. 2021[38]; Tang et al. 2021[17]	*Rezaii et al. 2019*[40]; *Haas et al. 2020*[41]; *Bilgrami et al. 2022*[42]; *Argolo et al. 2023*[22]	*Bedi et al. 2015*[43]	*Corcoran et al. 2018*[39]; *Bilgrami et al. 2022*[42]		*Corcoran et al. 2018*[39]; *Bilgrami et al. 2022*[42]	Bedi et al. 2015[43]; Tang et al. 2023b[26]
POS: adj	12	*Argolo et al. 2023*[22]	*Corcoran et al. 2018*[39]; Tang et al. 2021[17]; Ziv et al. 2022[28]	*Haas et al. 2020*[41]; *Bilgrami et al. 2022*[42]	*Bedi et al. 2015*[43]; Mitchell et al. 2015[34]; *Rezaii et al. 2019*[40]	*Corcoran et al. 2018*[39]	*Argolo et al. 2023*[22]	*Corcoran et al. 2018*[39]	Bedi et al. 2015[43]; Tang et al. 2023b[26]; Sarzynska-Wawer et al. 2021[38]
POS: adv	6		Tang et al. 2021[17]; Ziv et al. 2022[28]	*Haas et al. 2020*[41]; *Argolo et al. 2023*[22]	Mitchell et al. 2015[34]				Sarzynska-Wawer et al. 2021[38]
POS: verb	9	Mitchell et al. 2015[34]	Ziv et al. 2022[28]	*Haas et al. 2020*[41]; Tang et al. 2021[17]; *Argolo et al. 2023*[22]	*Bedi et al. 2015*[43]; *Rezaii et al. 2019*[40]				Sarzynska-Wawer et al. 2021[38]; Tang et al. 2023a[25]
POS: pron	9	Mitchell et al. 2015[34]; Tang et al. 2021[17]	*Corcoran et al. 2018*[39]	*Haas et al. 2020*[41]; *Argolo et al. 2023*[22]	*Bedi et al. 2015*[43]; *Rezaii et al. 2019*[40]	*Corcoran et al. 2018*[39]	Jeong et al. 2023[31]	*Corcoran et al. 2018*[39]	Sarzynska-Wawer et al. 2021[38]
POS: coorconj	4			*Haas et al. 2020*[41]; Tang et al. 2021[17]; *Argolo et al. 2023*[22]					Tang et al. 2023b[26]

POS: subconj	5	Silva et al. 2023[44]		*Haas et al. 2020*[41]; Tang et al. 2021[17]; *Argolo et al. 2023*[22]					Tang et al. 2023b[26]
POS: noun	7			*Haas et al. 2020*[41]; Tang et al. 2021[17]; Ziv et al. 2022[28]; *Argolo et al. 2023*[22]	*Bedi et al. 2015*[43]; Mitchell et al. 2015[34]				Sarzynska-Wawer et al. 2021[38]
ambiguous pronouns, referential failures	4	Iter et al. 2018[1]; Just et al. 2020[6]	*Morgan et al. 2021*[8]		*Nettekoven et al. 2023*[13]	Iter et al. 2018[1]	Iter et al. 2018[1]		
sentence / unit length	16		Iter et al. 2018[1]; *Morgan et al. 2021*[8]; *Spencer et al. 2021*[9]; Tang et al. 2021[17]; *Bilgrami et al. 2022*[42]; Silva et al. 2023[44]; *Nettekoven et al. 2023*[13]; Schneider et al. 2023[20]	Liang et al. 2022[11]; *Gupta et al. 2018*[15]; *Haas et al. 2020*[41]; *Morgan et al. 2021*[8]		Xu et al. 2020[45]; *Bilgrami et al. 2022*[42]	Liebenthal et al. 2023[24]; *Bilgrami et al. 2022*[42]; Silva et al. 2023[44]; Jeong et al. 2023[31]		Bedi et al. 2015[43]; Tang et al. 2023b[26]
sentence count	7	*Morgan et al. 2021*[8]	Iter et al. 2018[1]	*Gupta et al. 2018*[15]; Ryazanskaya & Khodyakova 2020[46]; *Morgan et al. 2021*[8]; Schneider et al. 2023[20]	Tang et al. 2021[17]		Jeong et al. 2023[31]		

SUPPLEMENTARY TABLE 2: Summary of the results reported for the syntactic methods used in the reviewed papers.

"+" indicates a significant group difference with higher values in the patient population. "-" indicates a significant group difference with lower values in the patient population. "!" indicates the absence of significant differences in the metric tested. "NR" indicates that the metric is used but results are not reported. "Corr. Symptoms" indicates a significant correlation with symptom severity in any direction. "No Corr. Symptoms" indicates the absence of significant correlation with symptom severity in any direction. "Corr. Ling. Scale" indicates a significant correlation with a linguistic symptom scale in any direction. "Classifier Feature" indicates that the metric is used as a feature in a classifier or latent analysis.

The studies on clinical high-risk populations are shown in italics. “POS” is a part-of-speech prefix. “det” stands for determiners and determiner pronouns. “adj” stands for adjectives. “pron” stands for pronoun. “adv” stands for adverb.

2.3. Graph-Based metrics

**Graph-Based metric**	**Count**	**-**	**!**	**Corr. Ling. Scale**	**Corr. Symptoms**	**No Corr. Symptoms**	**Classifier Feature**
number of nodes	4	Nikzad et al. 2022[47]	Mota et al. 2012[14]; *Nettekoven et al. 2023*[13]*			Mota et al. 2012[14]	
number of edges	6	Mota et al. 2014[2]; Mota et al. 2016[48]; Mota et al. 2017[49]; Nikzad et al. 2022[47]	Mota et al. 2012[14]; *Nettekoven et al. 2023*[13]*		Mota et al. 2014[2]; Nikzad et al. 2022[47]	Mota et al. 2012[14]	
LCC / median CC	11	Mota et al. 2014[2]; Mota et al. 2016[48]; Mota et al. 2017[49]; *Morgan et al. 2021*[8]; *Spencer et al. 2021*[9]; Nikzad et al. 2022[47]; Nettekoven et al. 2023[13]	*Morgan et al. 2021*[8]; *Spencer et al. 2021*[9]; *Nettekoven et al. 2023*[13]*; *Argolo et al. 2023*[22]	*Morgan et al. 2021*[8]; *Spencer et al. 2021*[9]; *Nettekoven et al. 2023*[13]**	Mota et al. 2014[2]; Mota et al. 2016[48]	Mota et al. 2012[14]; *Nettekoven et al. 2023*[13]	Mota et al. 2017[49]; Mota et al. 2023[35]
LSC	11	Mota et al. 2014[2]; Mota et al. 2016[48]; Mota et al. 2017[49]; *Morgan et al. 2021*[8]; *Spencer et al. 2021*[9]	*Morgan et al. 2021*[8]; *Spencer et al. 2021*[9]; Nikzad et al. 2022[47]; *Argolo et al. 2023*[22]*	*Morgan et al. 2021*[8]; *Spencer et al. 2021*[9]; Nikzad et al. 2022[47]	Mota et al. 2014[2]; Mota et al. 2016[48]; Nikzad et al. 2022[47]	Mota et al. 2012[14]; *Argolo et al. 2023*[22]*	Mota et al. 2017[49]; Mota et al. 2023[35]; *Argolo et al. 2023*[22]; Tang et al. 2023a[25]
LCCz/LCCr	5	*Morgan et al. 2021*[8]; *Spencer et al. 2021*[9]	Mota et al. 2016[48]; Mota et al. 2017[49]; *Morgan et al. 2021*[8]; *Spencer et al. 2021*[9]	*Morgan et al. 2021*[8]; *Spencer et al. 2021*[9]	Mota et al. 2016[48]		
LSCz/LSCr	5	Mota et al. 2016[48]; Mota et al. 2017[49]; *Morgan et al. 2021*[8]; *Spencer et al. 2021*[9]; Nikzad et al. 2022[47]	*Morgan et al. 2021*[8]; *Spencer et al. 2021*[9]	*Morgan et al. 2021*[8]; *Spencer et al. 2021*[9]; Nikzad et al. 2022[47]	Mota et al. 2016[48]; Nikzad et al. 2022[47]		Mota et al. 2017[49]; Mota et al. 2023[35]
ASP	6	Mota et al. 2014[2]	Mota et al. 2012[14]; Tang et al. 2021[17]; Nikzad et al. 2022[47]; *Argolo et al. 2023*[22]		Nikzad et al. 2022[47]	Mota et al. 2012[14]	*Argolo et al. 2023*[22]; Tang et al. 2023a[25]

density	5	Nikzad et al. 2022[47]	Mota et al. 2012[14]; Mota et al. 2014[2]; *Argolo et al. 2023*[22]*	Nikzad et al. 2022[47]	Nikzad et al. 2022[47]	Mota et al. 2012[14]	*Argolo et al. 2023*[22]; Tang et al. 2023a[25]
diameter	5	Mota et al. 2014[2]	Mota et al. 2012[14]; Nikzad et al. 2022[47]	Nikzad et al. 2022[47]		Mota et al. 2012[14]	Tang et al. 2023a[25]
ATD	4	Mota et al. 2014[2]	Mota et al. 2012[14]*			Mota et al. 2012[14]	Tang et al. 2023a[25]
AWD	1	Nikzad et al. 2022[47]		Nikzad et al. 2022[47]			
Largest Clique	2						Tang et al. 2023a[25]; Tang et al. 2023b[26]
clustering coefficient	1						Tang et al. 2023b[26]

SUPPLEMENTARY TABLE 3: Summary of the results reported for the graph-based metrics used in the reviewed papers.
"-" indicates a significant group difference with lower values in the patient population. "!" indicates the absence of significant differences in the metric tested. "NR" indicates that the metric is used but results are not reported. "Corr. Symptoms" indicates a significant correlation with symptom severity in any direction. "No Corr. Symptoms" indicates the absence of significant correlation with symptom severity in any direction. "Corr. Ling. Scale" indicates a significant correlation with a linguistic symptom scale in any direction. "Classifier Feature" indicates that the metric is used as a feature in a classifier or latent analysis.
The studies on clinical high-risk populations are shown in italics. Some studies report both for SZ and CHR and are reported twice.
"*" means the metric was insignificant after corrections for verbosity. "**" means the metric was insignificant after correcting for multiple comparisons.
"LCC" stands for the largest connected component. "CC" stands for a connected component. "LSC" stands for the largest strongly connected component. "LCCz/LCCr" stands for z-score or randomness of the largest connected component. "LSCz/LSCr" stands for z-score or randomness of the largest strongly connected component. "ASP" stands for the average shortest path. "ATD" stands for average total degree. "AWD" stands for average weighted degree.

2.4. LM-based metrics

Formulae for LM-based metrics are listed in Supplementary Table 10.

**Word-embedding-based metric**	**Count**	**+**	**-**	**!**	**Corr. Ling. Scale**	**Corr. Symptoms**	**No Corr. Symptoms**
Moving Window Coherence	8	Panicheva & Litvinova 2019[7] (min); Voppel et al. 2021[10] (var); Alonso-Sánchez et al. 2022[18]; Alonso-Sánchez et al. 2023[19]; Voppel et al. 2023[27] (var)	Bar et al. 2019[50]; Panicheva & Litvinova 2019[7](max); Parola et al. 2023[12] (Ch)	Doré 2019[4]; Parola et al. 2023[12] (D, G)			
Word-Based Coherence	5	Parola et al. 2023[12] (D)	Bar et al. 2019[50]; Parola et al. 2023[12] (Ch, G)	Liebenthal et al. 2023[24]; *Argolo et al. 2023*[22]	Xu et al. 2020[45]; Xu et al. 2022[51]		
K-Inter Word Similarity	3		*Corcoran et al. 2018*[39]	Parola et al. 2023[12] (?); *Argolo et al. 2023*[22]			*Corcoran et al. 2018*[39]
All word Similarity	3			Alonso-Sánchez et al. 2022[18]; Liebenthal et al. 2023[24]; Alonso-Sánchez et al. 2023[19]		Alonso-Sánchez et al. 2022[18]	
Vector Magnitude	2			*Rezaii et al. 2019*[40]; Liebenthal et al. 2023[24]			
Word-Based Centroid Global Coherence & Word-Based Cumulative Centroid Global	2				Xu et al. 2020[45]; Xu et al. 2022[51]		

Coherence							

Supplementary Table 4: Summary of the results reported for the word embedding-based metrics used in the reviewed papers. "-" indicates a significant group difference with lower values in the patient population. "!" indicates the absence of significant differences in the metric tested. "Corr. Symptoms" indicates a significant correlation with symptom severity in any direction. "No Corr. Symptoms" indicates the absence of significant correlation with symptom severity in any direction. "Corr. Ling. Scale" indicates a significant correlation with a linguistic symptom scale in any direction.
The studies on clinical high risk populations are shown in italics. Some studies report both for several languages - "Ch" stands for Chinese, "D" - for Danish, and "G" - for German. "min" indicates that minimum values are reported, "max" stands for maximum values, and "var" - for variance.

**Phrase-embeddin-based metric**	**Count**	**+**	**-**	**!**	**NR**	**Corr. Ling. Scale**	**Corr. Symptoms**	**No Corr. Symptoms**	**Classifier Feature**
(First-Order) Coherence	22		Iter et al. 2018[1]; Just et al. 2019[5]; Ryazanskaya 2020[16]; *Morgan et al. 2021*[8]	*Haas et al. 2020*[41]; Just et al. 2020[6]; *Hitczenko et al. 2021*[30]; *Bilgrami et al. 2022*[42]; Parola et al. 2023[12]	*Nettekoven et al. 2023*[13]	Xu et al. 2020[45]; Xu et al. 2022[51]; *Bilgrami et al. 2022*[42]	Ryazanskaya 2020[16]; Just et al. 2023[52]	*Bedi et al. 2015*[43]; Iter et al. 2018[1]; *Haas et al. 2020*[41]; *Hitczenko et al. 2021*[30]; Parola et al. 2023[12] (?); *Bilgrami et al. 2022*[42]	*Bedi et al. 2015*[43]; *Rosenstein et al. 2015*[32]; Iter et al. 2018[1]; Just et al. 2020[6]; Ryazanskaya & Khodyakova 2020[46]; Sarzynska-Wawer et al. 2021[38]; Tang et al. 2023a[25]; Tang et al. 2023b[26]
Second order Coherence	3		Parola et al. 2023[12]						*Bedi et al. 2015*[43]; Sarzynska-Wawer et al. 2021[38]
Repetitiveness	1			*Morgan et al. 2021*[8]					
Group Global Coherence	4		Ryazanskaya 2020[16]			Elvevåg et al. 2007[53]; Elvevåg et al. 2010[54]; Ryazanskaya &	Ryazanskaya 2020[16]		

						Khodyakova 2020[46]			
Gold Standard Global Coherence	3		*Morgan et al. 2021*[8]		*Nettekoven et al. 2023*[13]	Ryazanskaya & Khodyakova 2020[46]			
Centroid Global Coherence	4		Ryazanskaya 2020[16]			Xu et al. 2020[45]; Xu et al. 2022[51]	Ryazanskaya 2020[16]; Just et al. 2023[52]		
Cumulative Centroid Global Coherence	3		Ryazanskaya 2020[16]			Xu et al. 2020[45]; Xu et al. 2022[51]	Ryazanskaya 2020[16]		
Slope Tangentiality	9	Iter et al. 2018[1]; Tang et al. 2021[17]		Koránová 2017[21]; Just et al. 2019[5]; Doré 2019[4]; *Hitczenko et al. 2021*[30]; *Morgan et al. 2021*[8]		Elvevåg et al. 2007[53]		Doré 2019[4]; *Hitczenko et al. 2021*[30]; *Morgan et al. 2021*[8]	
Q-similarity Tangentiality	4		*Morgan et al. 2021*[8]	Koránová 2017[21]; Just et al. 2019[5]	*Nettekoven et al. 2023*[13]				

SUPPLEMENTARY TABLE 5: Summary of the results reported for the phrase embedding-based metrics used in the reviewed papers.
"-" indicates a significant group difference with lower values in the patient population. "!" indicates the absence of significant differences in the metric tested. "Corr. Symptoms" indicates a significant correlation with symptom severity in any direction. "No Corr. Symptoms" indicates the absence of significant correlation with symptom severity in any direction. "Corr. Ling. Scale" indicates a significant correlation with a linguistic symptom scale in any direction.
The studies on clinical high-risk populations are shown in italics. "?" indicates that the information in the article only allows for a presumable placement of the article.

LM-feature-based metric	Count	+	-	!	NR	Corr. Ling. Scale	Corr. Symptoms	No Corr. Symptoms	Classifier Feature
Perplexity / Surprisal	5	Srivastava et al. 2022a[55]		Mitchell et al. 2015[34]; *Srivastava et al. 2022a[55];* Jeong et al. 2023[31]		Jeong et al. 2023[31]	Vail et al. 2018[36]; Girard et al. 2022[37]; Jeong et al. 2023[31]		
Next-Sentence Prediction	3			*Hitczenko et al. 2021[30];* Tang et al. 2021[17]		Jeong et al. 2023[31]	Jeong et al. 2023[31]	Tang et al. 2021[17]	
Non-Fine-Tuned Classifier	3								Elvevåg et al. 2010[54]; *Rosenstein et al. 2015[32]*; *Srivastava et al. 2022b[56]*
Fine-Tuned Classifier	3								Wouts et al. 2021[57]; Aich et al. 2022[29]; Shriki et al. 2022[58]

Supplementary Table 6: Summary of the results reported for the LM-based feature metrics used in the reviewed papers.
"+" indicates a significant group difference with higher values in the patient population. "!" indicates the absence of significant differences in the metric tested. "Corr. Symptoms" indicates a significant correlation with symptom severity in any direction. "No Corr. Symptoms" indicates the absence of significant correlation with symptom severity in any direction. "Corr. Ling. Scale" indicates a significant correlation with a linguistic symptom scale in any direction.
The studies on clinical high-risk populations are shown in italics.

Cross-sentence aggregation	Count	Papers
mean	28	Bedi et al. 2015[43]; Mitchell et al. 2015[34]; Corcoran et al. 2018[39]; Just et al. 2019[5]; Bar et al. 2019[50]; Panicheva & Litvinova 2019[7]; Doré 2019[4]; Haas et al. 2020[41]; Just et al. 2020[6]; Ryazanskaya 2020[16]; Xu et al. 2020[45]; Voppel et al. 2021[10]; Hitczenko et al. 2021[30]; Morgan et al. 2021[8]; Sarzynska-Wawer et al. 2021[38]; Alonso-Sánchez et al. 2022[18]; Corona-Hernández et al. 2022[59]; Voppel et al. 2023[27]; Alonso-Sánchez et al. 2023[19]; Girard et al. 2022[37]; Srivastava et al. 2022[55]; Srivastava et al. 2022[56]; Jeong et al. 2023[31]; Liebenthal et al. 2023[24]; Nettekoven et al. 2023[13]; Tang et al. 2023a[25]; Tang et al. 2023b[26]; Just et al. 2023[52]; Argolo et al. 2023[22]
minimum	14	Bedi et al. 2015[43]; Iter et al. 2018[1]; Corcoran et al. 2018[39]; Panicheva & Litvinova 2019[7]; Haas et al. 2020[41]; Ryazanskaya 2020[16]; Xu et al. 2020[45]; Morgan et al. 2021[8]; Sarzynska-Wawer et al. 2021[38]; Voppel et al. 2021[10]; Bilgrami et al. 2022[42]; Corona-Hernández et al. 2022[59]; Xu et al. 2022[51];

		Voppel et al. 2023[27]
maximum	11	Bedi et al. 2015[43]; Corcoran et al. 2018[39]; Panicheva & Litvinova 2019[7]; Haas et al. 2020[41]; Ryazanskaya 2020[16]; Morgan et al. 2021[8]; Bilgrami et al. 2022[42]; Corona-Hernández et al. 2022[59]; Voppel et al. 2023[27]; Just et al. 2023[52]; Argolo et al. 2023[22]
SD / variance	9	Bedi et al. 2015[43]; Corcoran et al. 2018[39]; Panicheva & Litvinova 2019[7]; Haas et al. 2020[41]; Hitczenko et al. 2021[30]; Sarzynska-Wawer et al. 2021[38]; Voppel et al. 2021[10]; Corona-Hernández et al. 2022[59]; Voppel et al. 2023[27]
median	4	Bedi et al. 2015[43]; Sarzynska-Wawer et al. 2021[38]; Corona-Hernández et al. 2022[59]; Parola et al. 2023[12]
percentile	2	Corcoran et al. 2018[39]; Panicheva & Litvinova 2019[7]
IQR	1	Parola et al. 2023[12]

Supplementary Table 7: Cross-sentence aggregation methods used for LM-based metrics in the reviewed papers. "SD" stands for standard deviation.

LM	Count	Papers
lsa	10	Elvevåg et al. 2007[53]; Elvevåg et al. 2010[54]; Bedi et al. 2015[43]; Rosenstein et al. 2015[32]; Iter et al. 2018[1]; Xu et al. 2020[45]; Haas et al. 2020[41]; Hitczenko et al. 2021[30]; Tang et al. 2023a[25]; Tang et al. 2023b[26]
w2v	24	Kořánová 2017[21]; Iter et al. 2018[1]; Rezaii et al. 2019[40]; Just et al. 2019[5]; Bar et al. 2019[50]; Panicheva & Litvinova 2019[7]; Doré 2019[4]; Ryazanskaya 2020[16]; Ryazanskaya & Khodyakova 2020[46]; Xu et al. 2020[45]; Voppel et al. 2021[10]; Hitczenko et al. 2021[30]; Morgan et al. 2021[8]; Sarzynska-Wawer et al. 2021[38]; Corona-Hernández et al. 2022[59]; Parola et al. 2023[12]; Xu et al. 2022[51]; Argolo et al. 2023[22]; Just et al. 2023[52]; Liebenthal et al. 2023[24]; Nettekoven et al. 2023[13]; Tang et al. 2023a[25]; Tang et al. 2023b[26]; Voppel et al. 2023[27]
glove	8	Iter et al. 2018[1]; Just et al. 2020[6]; Hitczenko et al. 2021[30]; Alonso-Sánchez et al. 2022[18]; Alonso-Sánchez et al. 2023[19]; Tang et al. 2023a[25]; Tang et al. 2023b[26]; Just et al. 2023[52]
bert	10	Ryazanskaya 2020[16]; Hitczenko et al. 2021[30]; Tang et al. 2021[17]; Wouts et al. 2021[57]; Aich et al. 2022[29]; Bilgrami et al. 2022[42]; Srivastava et al. 2022a[55]; Shriki et al. 2022[58]; Xu et al. 2022[51]; Jeong et al. 2023[31]
elmo	4	Ryazanskaya 2020[16]; Hitczenko et al. 2021[30]; Sarzynska-Wawer et al. 2021[38]; Srivastava et al. 2022b[56]
sent2vec	3	Iter et al. 2018[1]; Just et al. 2019[5]; Hitczenko et al. 2021[30]
trigram	3	Mitchell et al. 2015[34]; Vail et al. 2018[36]; Girard et al. 2022[37]

backoff		

SUPPLEMENTARY TABLE 8: Models used to obtain the LM-based metrics in the reviewed papers.

**Within sentence averaging**	**Count**	**Papers**
mean	23	Elvevåg et al. 2007[53]; Elvevåg et al. 2010[54]; Bedi et al. 2015[43]; Rosenstein et al. 2015[32]; Corcoran et al. 2018[39]; Rezaii et al. 2019[40]; Bar et al. 2019[50]; Panicheva & Litvinova 2019[7]; Doré 2019[4]; Haas et al. 2020[41]; Voppel et al. 2021[10]; Hitczenko et al. 2021[30]; Tang et al. 2021[17]; Sarzynska-Wawer et al. 2021[38]; Corona-Hernández et al. 2022[59]; Alonso-Sánchez et al. 2022[18]; Parola et al. 2023[12]; Alonso-Sánchez et al. 2023[19]; Just et al. 2023[52]; Liebenthal et al. 2023[24]; Tang et al. 2023a[25]; Tang et al. 2023b[26]; Voppel et al. 2023[27]
tf-idf	8	Iter et al. 2018[1]; Just et al. 2019[5]; Just et al. 2020[6]; Ryazanskaya & Khodyakova 2020[46]; Xu et al. 2020[45]; Hitczenko et al. 2021[30]; Xu et al. 2022[51]; Tang et al. 2023b[26]
sif	6	Iter et al. 2018[1]; Just et al. 2019[5]; Ryazanskaya 2020[16]; Hitczenko et al. 2021[30]; Morgan et al. 2021[8]; Nettekoven et al. 2023[13]
sum	1	Xu et al. 2020[45]

SUPPLEMENTARY TABLE 9: Sentence averaging methods used for word embedding-based metrics in the reviewed paper

2.5. Formulae for the discussed LM-based metrics

Metric Name	Definition	Introduced
(First-Order) Coherence	$agg_{i=1}{}^{N}(cossim(S_i, S_{i+1}))$	Bedi et al. 2015[43]
Second-Order Coherence	$agg_{i=1}{}^{N-1}(cossim(S_i, S_{i+2}))$	Bedi et al. 2015[43]
Repetitiveness	$max_{i,j=1;\, i\neq j}{}^{N}(cossim(S_i, S_j))$	Morgan et al., 2021[8]
Moving Window Coherence	$agg_{i=1}{}^{M-k}(mean(cossim(W_x, W_y)))$ for $0 < y - x \leq k; i \leq x, y < M$	Bar et al. 2019[50] Panicheva & Litvinova 2019[7]
K-Inter Word Similarity	$agg_{i=1}{}^{M-k}(mean(cossim(W_i, W_{i+k})))$	Corcoran et al. 2018[39]
Group Global Coherence	$cossim(R_a, mean_{b=1}{}^{Q}(R_b))$	Elvevåg et al. 2007[53]
Gold Standard Global Coherence	$cossim(R_a, R_{gold})$	Ryazanskaya 2020[16]
Centroid Global Coherence	$agg_{i=1}{}^{N}(cossim(S_i, mean_{j=1}{}^{N}(S_j)))$	Xu et al. 2020[45]
Cumulative Centroid Global Coherence	$agg_{i=1}{}^{N}(cossim(S_i, mean_{j=1}{}^{i}(S_j)))$	Xu et al. 2020[45]
Slope Tangentiality	$agg_{i=1}{}^{N}(slope_{i=1}{}^{N}(cossim(S_q, S_i)))$	Elvevåg et al. 2007[53]
Q-similarity Tangentiality	$agg_{i=1}{}^{N}(cossim(S_q, S_i))$	Kořánová 2017[21]

SUPPLEMENTARY TABLE 10: Formulae for the discussed embedding-based methods.
S - sentence embedding; W - word embedding; R - response embedding; $cossim$ - cosine similarity; agg - aggregation scheme (such as mean, max, min, or median); $slope$- slope of a linear regression function; S_q - question embedding; R_{gold} - gold standard description embedding; M - number of words; N - number of sentences; Q - number of people in the group.

S3 Supplementary Table summarizing metric performance

3.1. Top 5 metrics by absolute performance averaged across tasks

Scale	Subscale	German	Russian
PANSS	total	number of edges, LCC, number of nodes, LSC, POS rate: PART	**word count**, *number of edges, LSC, parallel edges, LCC*
PANSS	negative	number of edges, LCC, number of nodes, LSC, LTR	***mean sent len***, *w2v tf scoh, w2v mean scoh, POS rate: NOUN,* ***word count***
PANSS	general psychopathology	LCC, number of nodes, number of edges, LSC, POS rate: PART	**word count**, ***sent count***, *number of edges, loops of size 1, LCC*
PANSS	positive	*w2v tf lcoh, w2v mean lcoh, POS rate: AUX, GloVe tf scoh, POS rate: PART*	parallel edges, *LSC*, *number of edges*, *LTR*, *LCC*
t-test	psychosis vs control	max sent len, std sent len, **mean sent len**, **word count**, LCC	**word count**, LTR, number of edges, LCC, number of nodes
t-test	depression vs control		LTR, **word count**, **sent count**, number of edges, LCC
t-test	psychosis vs depression		POS rate: PART, POS rate: PROPN, *MALTR*, *POS rate: DET*, *LSC*

SUPPLEMENTARY TABLE 11: Lists of the 5 metrics with the highest absolute performance as measured by Pearson's r correlation coefficient with the PANSS subscales and t-test statistic on the German and Russian datasets averaged across tasks. The metrics are presented from best to worst. The baselines are highlighted in bold. For the metrics highlighted in italics, the 95% confidence intervals obtained with bootstrapping intersected zero.

Supplementary References

1. Iter, D., Yoon, J. & Jurafsky, D. Automatic detection of incoherent speech for diagnosing schizophrenia. in *Proc. 5th Workshop on Computational Linguistics and Clinical Psychology* 136–146 (2018).
2. Mota, N. B., Furtado, R., Maia, P. P. C., Copelli, M. & Ribeiro, S. Graph analysis of dream reports is especially informative about psychosis. *Sci. Rep.* **4**, 3691 (2014).
3. Willits, J. A., Rubin, T., Jones, M. N., Minor, K. S. & Lysaker, P. H. Evidence of disturbances of deep levels of semantic cohesion within personal narratives in schizophrenia. *Schizophr. Res.* **197**, 365–369 (2018).

4. Doré, M.N. *Quantification of Coherence in Spoken Language as an Indicator for the Schizophrenia Spectrum Disorder*. BSc thesis, Utrecht Univ. (2019).

5. Just, S. *et al.* Coherence models in schizophrenia. in *Proceedings of the Sixth Workshop on Computational Linguistics and Clinical Psychology* 126–136 (2019).

6. Just, S. A. *et al.* Modeling incoherent discourse in non-affective psychosis. *Front. Psychiatry* **11,** 846 (2020).

7. Panicheva, P. & Litvinova, T. Semantic coherence in schizophrenia in Russian written texts. in *25th Conference of Open Innovations Association (FRUCT)* 241–249 (2019).

8. Morgan, S. E. *et al.* Natural language processing markers in first episode psychosis and people at clinical high-risk. *Transl. Psychiatry* **11**, 630 (2021).

9. Spencer, T. J. *et al*. Lower speech connectedness linked to incidence of psychosis in people at clinical high risk. *Schizophr. Res.* **228**, 493–501 (2021).

10. Voppel, A. E., de Boer, J. N., Brederoo, S. G., Schnack, H. G., & Sommer, I. E. C.. Quantified language connectedness in schizophrenia-spectrum disorders. *Psychiatry Res.* **304**, 114130 (2021).

11. Liang, L. *et al.* Widespread cortical thinning, excessive glutamate and impaired linguistic functioning in schizophrenia: a cluster analytic approach. *Front. Hum. Neurosci.* **16,** 954898 (2022).

12. Parola, A. *et al.* Speech disturbances in schizophrenia: assessing cross-linguistic generalizability of NLP automated measures of coherence. *Schizophr. Res.* **259**, 59–70 (2023).

13. Nettekoven, C. R. *et al.* Semantic speech networks linked to formal thought disorder in early psychosis. *Schizophr. Bull.* **49**, S142–S152 (2023).

14. Mota, N. B. *et al.* Speech graphs provide a quantitative measure of thought disorder in psychosis. *PLoS ONE* **7**, e34928 (2012).

15. Gupta, T., Hespos, S. J., Horton, W. S., & Mittal, V. A.. Automated analysis of written narratives reveals abnormalities in referential cohesion in youth at ultra high risk for psychosis. *Schizophr.*

Res. **192**, 82–88 (2018).

16. Ryazanskaya, G. *Automated Assessment of Discourse Coherence in Schizophrenia and Schizoaffective Disorder*. BSc thesis, Higher School of Economics (2020).
17. Tang, S. X. *et al.* Natural language processing methods are sensitive to sub-clinical linguistic differences in schizophrenia spectrum disorders. *npj Schizophr.* **7**, 25 (2021).
18. Alonso-Sánchez, M. F. *et al*. Progressive changes in descriptive discourse in first episode schizophrenia: a longitudinal computational semantics study. *Schizophrenia* **8**, 36 (2022).
19. Alonso-Sánchez, M.F., Limongi, R., Gati, J. & Palaniyappan, L. Language network self-inhibition and semantic similarity in first-episode schizophrenia: a computational-linguistic and effective connectivity approach. *Schizophr. Res.* **259**, 97–103 (2023).
20. Schneider, K. *et al.* Syntactic complexity and diversity of spontaneous speech production in schizophrenia spectrum and major depressive disorders. *Schizophrenia* **9**, 35 (2023).
21. Kořánová, N. *Analyzing Coherence in Spontaneous Speech of Schizophrenic Patients*. MSc thesis, Univ. Potsdam (2017).
22. Argolo, F.C. *et al.* Burnishing the blueprint of speech assessment with natural language processing: methods to characterize subtle impairments on individuals in at-risk mental states from a large urban population. Preprint at PsyArXiv https://doi.org/10.31234/osf.io/epgfy (2023).
23. Minor, K. S., Lundin, N. B., Myers, E. J., Fernández-Villardón, A. & Lysaker, P. H. Automated measures of speech content and speech organization in schizophrenia: test-retest reliability and generalizability across demographic variables. *Psychiatry Res.* **320**, 115048 (2023).
24. Liebenthal, E. *et al.* Linguistic and non-linguistic markers of disorganization in psychotic illness. *Schizophr. Res.* **259**, 111–120 (2023).
25. Tang, S. X. *et al*. Clinical and computational speech measures are associated with social cognition in schizophrenia spectrum disorders. *Schizophr. Res.* **259**, 28–37 (2023).
26. Tang, S. X. *et al.* Latent factors of language disturbance and relationships to quantitative speech features. *Schizophr. Bull.* **49**, S93–S103 (2023).

27. Voppel, A. E., de Boer, J. N., Brederoo, S. G., Schnack, H. G. & Sommer, I. E. C. Semantic and acoustic markers in schizophrenia-spectrum disorders: a combinatory machine learning approach. *Schizophr. Bull.* **49**, S163–S171 (2023).

28. Ziv, I. *et al.* Morphological characteristics of spoken language in schizophrenia patients – an exploratory study. *Scand. J. Psychol.* **63,** 91–99 (2022).

29. Aich, A. *et al.* Towards intelligent clinically-informed language analyses of people with bipolar disorder and schizophrenia. in *Findings of the Association for Computational Linguistics: EMNLP 2022* 2871–2887 (2022).

30. Hitczenko, K., Mittal, V. A. & Goldrick, M. Understanding language abnormalities and associated clinical markers in psychosis: the promise of computational methods. *Schizophr. Bull.* **47**, 344–362 (2021).

31. Jeong, L. *et al.* Exploring the use of natural language processing for objective assessment of disorganized speech in schizophrenia. *Psychiatr. Res. Clin. Pract.* **5**, 84–92 (2023).

32. Rosenstein, M., Foltz, P. W., DeLisi, L. E., & Elvevåg, B. Language as a biomarker in those at high-risk for psychosis. *Schizophr. Res.* **165**, 249–250 (2015).

33. Kramov, A. Evaluating text coherence based on the graph of the consistency of phrases to identify symptoms of schizophrenia. Preprint at arXiv https://doi.org/10.48550/arXiv.2005.03008 (2020).

34. Mitchell, M., Hollingshead, K. & Coppersmith, G. Quantifying the language of schizophrenia in social media. in *Proc. 2nd Workshop on Computational Linguistics and Clinical Psychology* 11–20 (2015).

35. Mota, N. B., *et al*. Happy thoughts: what computational assessment of connectedness and emotional words can inform about early stages of psychosis. *Schizophr. Res.* **259**, 38–47 (2023).

36. Vail, A.K., Liebson, E., Baker, J.T. & Morency, L.-P. Toward objective, multifaceted characterization of psychotic disorders: lexical, structural, and disfluency markers of spoken language. in *Proceedings of the 20th ACM International Conference on Multimodal Interaction*

170–178 (2018).

37. Girard, J. M. *et al.* Computational analysis of spoken language in acute psychosis and mania. *Schizophr. Res.* **245**, 97–115 (2022).

38. Sarzynska-Wawer, J. *et al*. Detecting formal thought disorder by deep contextualized word representations. *Psychiatry Res.* **304**, 114135 (2021).

39. Corcoran, C. M. *et al.* Prediction of psychosis across protocols and risk cohorts using automated language analysis. *World Psychiatry* **17**, 67–75 (2018).

40. Rezaii, N., Walker, E. & Wolff, P. A machine learning approach to predicting psychosis using semantic density and latent content analysis. *npj Schizophr.* **5**, 9 (2019).

41. Haas, S.S. *et al.* Linking language features to clinical symptoms and multimodal imaging in individuals at clinical high risk for psychosis. *Eur. Psychiatry* **63,** e72 (2020).

42. Bilgrami, Z. R., *et al*. Construct validity for computational linguistic metrics in individuals at clinical risk for psychosis: associations with clinical ratings. *Schizophr. Res.* **245**, 90–96 (2022).

43. Bedi, G. *et al.* Automated analysis of free speech predicts psychosis onset in high-risk youths. *npj Schizophr.* **1**, 15030 (2015).

44. Silva, A. M. *et al.* Syntactic complexity of spoken language in the diagnosis of schizophrenia: a probabilistic Bayes network model. *Schizophr. Res.* **259**, 88–96 (2023).

45. Xu, W., Portanova, J., Chander, A., Ben-Zeev, D. & Cohen, T. The centroid cannot hold: comparing sequential and global estimates of coherence as indicators of formal thought disorder. *AMIA Annu. Symp. Proc.* **2020,** 1315 (2020).

46. Ryazanskaya, G. & Khudyakova, M. Automated analysis of discourse coherence in schizophrenia: approximation of manual measures. in *LREC 2020 Language Resources and Evaluation Conference* 98–101 (2020).

47. Nikzad, A. H. *et al.* Who does what to whom? Graph representations of action-predication in speech relate to psychopathological dimensions of psychosis. *Schizophrenia* **8**, 58 (2022).

48. Mota, N.B., Copelli, M. & Ribeiro, S. Quantifying word salad: the structural randomness of

verbal reports predicts negative symptoms and schizophrenia diagnosis 6 months later. Preprint at arXiv https://doi.org/10.48550/arXiv.1610.08566 (2016).

49. Mota, N.B., Copelli, M. & Ribeiro, S. Thought disorder measured as random speech structure classifies negative symptoms and schizophrenia diagnosis 6 months in advance. *npj Schizophr.* **3,** 18 (2017).

50. Bar, K. *et al.* Semantic characteristics of schizophrenic speech. in *Proc. 6th Workshop on Computational Linguistics and Clinical Psychology* 84–93 (2019).

51. Xu, W. *et al.* Fully automated detection of formal thought disorder with time-series augmented representations for detection of incoherent speech (TARDIS). *J. Biomed. Inform.* **126,** 103998 (2022).

52. Just, S. A. *et al.* Validation of natural language processing methods capturing semantic incoherence in the speech of patients with non-affective psychosis. *Front. Psychiatry* **14**, 1208856 (2023).

53. Elvevåg, B., Foltz, P. W., Weinberger, D. R. & Goldberg, T. E. Quantifying incoherence in speech: an automated methodology and novel application to schizophrenia. *Schizophr. Res.* **93**, 304–316 (2007).

54. Elvevåg, B., Foltz, P. W., Rosenstein, M., & DeLisi, L. E.. An automated method to analyze language use in patients with schizophrenia and their first-degree relatives. *J. Neurolinguistics* **23**, 270–284 (2010).

55. Srivastava, A. *et al.* Estimating self-disturbance in psychosis and its risk states using natural language processing analysis of open-ended interviews. *Biol. Psychiatry* **91,** S280 (2022).

56. Srivastava, A. *et al.* Increased metaphor production in open-ended speech samples of patients with prodromal and developed schizophrenia detected with NLP. *Biol. Psychiatry* **91**, S50 (2022).

57. Wouts, J. *et al.* belabBERT: a Dutch RoBERTa-based language model applied to psychiatric classification. Preprint at arXiv https://doi.org/10.48550/arXiv.2106.01091 (2021).

58. Shriki, Y., Ziv, I., Dershowitz, N., Harel, E. & Bar, K. Masking morphosyntactic categories to

evaluate salience for schizophrenia diagnosis. in *Proc. 8th Workshop on Computational Linguistics and Clinical Psychology* 148–157 (2022).

59. Corona-Hernández, H., de Boer, J. N., Brederoo, S. G., Voppel, A. E., & Sommer, I. E. C. Assessing coherence through linguistic connectives: analysis of speech in patients with schizophrenia-spectrum disorders. *Schizophr. Res.* **259**, 48–58 (2023).
